# Activating alternative transport modes in a multidrug resistance efflux pump to confer chemical susceptibility

**DOI:** 10.1038/s41467-022-35410-2

**Published:** 2022-12-10

**Authors:** Peyton J. Spreacker, Nathan E. Thomas, Will F. Beeninga, Merissa Brousseau, Colin J. Porter, Kylie M. Hibbs, Katherine A. Henzler-Wildman

**Affiliations:** 1 Department of Biochemistry, University of Wisconsin-Madison, Madison, WI 53703 USA; 2 National Magnetic Resonance Facility at Madison, University of Wisconsin-Madison, Madison, WI 53703 USA; 3 Present Address: Department of Chemistry and Biochemistry, University of California-San Diego, La Jolla, CA 92093 USA

**Keywords:** Membrane proteins, Permeation and transport, Bacteria, Electrophysiology

## Abstract

Small multidrug resistance (SMR) transporters contribute to antibiotic resistance through proton-coupled efflux of toxic compounds. Previous biophysical studies of the *E. coli* SMR transporter EmrE suggest that it should also be able to perform proton/toxin symport or uniport, leading to toxin susceptibility rather than resistance in vivo. Here we show EmrE does confer susceptibility to several previously uncharacterized small-molecule substrates in *E. coli*, including harmane. In vitro electrophysiology assays demonstrate that harmane binding triggers uncoupled proton flux through EmrE. Assays in *E. coli* are consistent with EmrE-mediated dissipation of the transmembrane pH gradient as the mechanism underlying the in vivo phenotype of harmane susceptibility. Furthermore, checkerboard assays show this alternative EmrE transport mode can synergize with some existing antibiotics, such as kanamycin. These results demonstrate that it is possible to not just inhibit multidrug efflux, but to activate alternative transport modes detrimental to bacteria, suggesting a strategy to address antibiotic resistance.

## Abstract (web summary)

Antibiotic resistance through multidrug efflux is a major hurdle in antibiotic development. Here, authors experimentally demonstrate that EmrE, a small multidrug resistance efflux pump from E. coli, can confer resistance or susceptibility depending on the small molecule substrate.

## Introduction

There is an urgent need to better understand the underlying mechanisms of antibiotic action and resistance. One mechanism by which bacteria survive antibiotic exposure is through the efflux of toxic compounds by promiscuous multidrug transporters. Most of these bacterial multidrug resistance transporters operate through an antiport mechanism wherein the efflux of toxic substrates is driven by the downhill import of H^+^ (or Na^+^). The small multidrug resistance (SMR) transporter, EmrE, has become an important model system for studying the mechanism of proton-coupled efflux and multidrug recognition because of its amenability to structural biology, biophysical experiments, and in vitro assays. The discovery that EmrE could perform coupled 2 H^+^: 1 substrate (toxin) antiport of a wide range of polyaromatic cations^1^ defined its function for many years. However, recent discoveries have highlighted the exceptional promiscuity of transporters in this family despite their small size. The SMR family was the first membrane protein discovered to have an unusual antiparallel homodimer topology; more recently part of the family was reclassified as toxic metabolite exporters rather than multidrug efflux pumps; and finally, NMR studies of EmrE indicate that this transporter should be capable of multiple modes of transport, not just proton-coupled antiport as required for antibiotic resistance^2–4^. Here we focus on the biological implications of alternative transport modes and whether it is possible to not just inhibit multidrug resistance efflux pumps to suppress their contribution to antibiotic resistance, but rather activate alternative transport modes that would be detrimental to bacterial metabolism, growth, and survival.

**Fig. 1:**
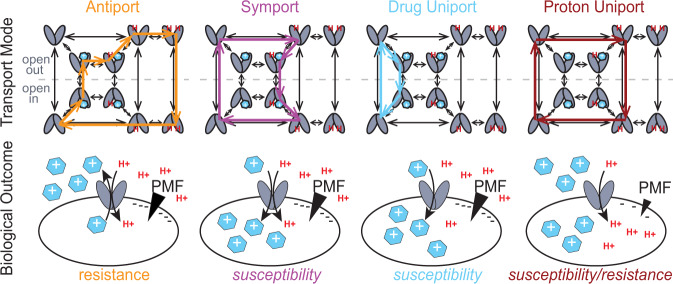
Different transport modes of EmrE result in different biological outcomes. The well-established coupled antiport of proton and drug (orange, top) by EmrE leads to drug resistance in vivo (orange, bottom). The Free Exchange Model suggests that EmrE should also be able to perform coupled symport (purple), or drug uniport (blue), either of which would lead to susceptibility rather than resistance in vivo. Proton uniport (maroon) will dissipate ∆pH and could lead to either resistance or susceptibility using an antibiotic adjuvant PMF dissipator with a known antibiotic. The most likely pathway depends on the relative rates of the microscopic steps in the transport cycle, including drug on- and off-rates and the rate of alternating access between open-in and open-out conformations in each state (apo, proton-bound, drug-bound, etc.). Thus, different substrates can lead to different dominant modes of transport and opposing biological outcomes in cells.

Traditional models of proton-coupled antiport focus on the key states and transitions needed for stoichiometric coupled antiport and assume that other states and transitions (leak pathways) contribute minimally to net transport because these alternative pathways would be deleterious *in the cell*. Recently, careful exploration of the states and transitions of EmrE using NMR revealed that this assumption is incorrect^4^. Expanding the mechanistic model to include all the observed states and transitions leads to a more complex free exchange model where proton/toxin symport, proton uniport, and toxin uniport are all theoretically possible in addition to the well-established proton/substrate antiport activity of EmrE (Fig. 1). The biological implications of these alternative transport pathways are significant. While H^+^-driven antiport results in toxin efflux and a resistance phenotype in vivo, proton-coupled symport or toxin uniport would result in active uptake of the toxic molecule into bacteria. Proton leak will rundown the proton motive force, disrupting bacterial energy metabolism, and is also likely to lead to a susceptibility phenotype in bacteria.

There is precedent for converting SMR transporter activity from conferring resistance to susceptibility in vivo by mutating the transporter^5^. The W63G-EmrE point mutant confers resistance to the clinical antibiotic erythromycin, but susceptibility to polyamine compounds^6^, confirming that both transport phenotypes are possible for a single transporter. Of greater biomedical relevance is whether it is possible to shift wild-type (WT) EmrE from its well-established resistance activity to activate alternative transport modes that would confer susceptibility (Fig. 1). There are a few examples of WT transporters utilizing different transport modes to optimize physiological outcomes for sugar uptake under changing external conditions^7,8^ or by preventing loss of acquired metals through back transport^9,10^. EmrE would represent a fundamentally different case where different modes of transport result in opposite biological outcomes of resistance versus susceptibility to toxic compounds.

In the case of EmrE, our prior biophysical studies and simulations show that 2 H^+^:1 toxin antiport is kinetically favored under physiological conditions for substrates to which EmrE is known to confer resistance^11^. This is consistent with the mechanistic requirements for driving toxin efflux by coupling to the proton motive force, which is inwardly directed in bacteria. However, we have also shown that the rates of key microscopic steps in the transport cycle vary by up to three orders of magnitude depending on which small molecule is being transported^12,13^. Rate changes on this scale have the potential to bias flux through alternative transport pathways and shift the balance of net transport^4^.

Here we experimentally test whether small-molecule substrates can activate alternative transport modes in WT-EmrE in a manner that negatively impacts bacterial metabolism and growth. Using an unbiased small-molecule phenotypic screen, we identify substrates to which EmrE confers resistance and substrates to which it confers susceptibility. Harmane is one of the substrates that most strongly activates susceptibility in vivo. We use an in vitro solid-supported membrane electrophysiology assay to show that harmane triggers uncontrolled proton leak through EmrE, defining the molecular mechanism underlying the susceptibility phenotype. The susceptibility phenotype observed in both bacterial growth and NADH production is consistent with harmane-gated proton leak causing run down of the transmembrane pH gradient. Additional in vivo growth assays show that bicarbonate suppresses the susceptibility phenotype caused by harmane and that harmane synergizes with kanamycin, both results as expected for selective dissipation of ∆pH by harmane-gated proton leak through EmrE. This work opens the possibility of developing small molecules to activate alternative transport pathways of multidrug transporters as an approach to target antibiotic resistance.

## Results

### An unbiased screen reveals previously unidentified substrates

**Fig. 2:**
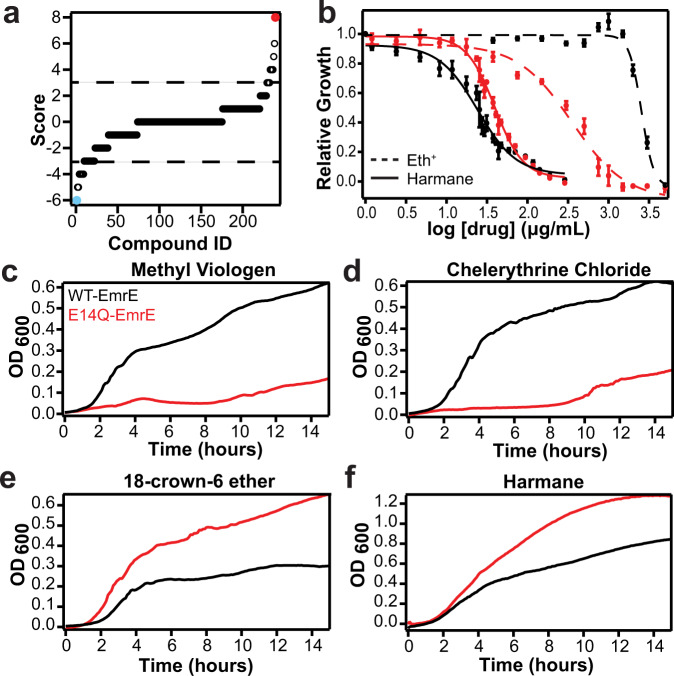
EmrE can confer either resistance or susceptibility in vivo. **a** Biolog phenotype microarray results were sorted by hit score for all 240 compounds in the screen (see Methods). Scores above +3 or below −3 are considered resistance or susceptibility hits, respectively, based on the differential between functional (WT) and nonfunctional (E14Q-EmrE). The strongest resistance hits (red) and susceptibility hits (cyan) were tested in growth assays (C-F). **b** IC_50_ curves of WT- (black) and E14Q-EmrE (red) are shown for ethidium bromide (dashed lines, resistance) and harmane (solid lines, susceptibility). *N* = 3 biologically independent bacterial colonies were examined over three independent experiments. Data were presented as mean value ± SD. Note that cells expressing WT-EmrE have a 40% lower IC_50_ value than cells expressing E14Q-EmrE in the presence of harmane. MG1655 ∆emrE *E. coli* expressing WT-EmrE (black) or E14Q-EmrE (red) were grown in the presence of **c** 0.5 mM methyl viologen (MV^2+^), **d** 0.05 mM chelerythrine chloride (CC), **e** 0.1 mM 18-crown-6-ether, or **f** 0.13 mM harmane. As expected, *E. coli* expressing WT-EmrE survived in the presence of MV^2+^ and CC (**c**, **d**), but *E. coli* expressing E14Q-EmrE did not, consistent with a resistance phenotype. In contrast, *E. coli* expressing nonfunctional, E14Q-EmrE had a higher OD_600_ at the stationary phase than *E. coli* expressing WT-EmrE in the presence of 18-crown-6-ether and harmane, (**e**, **f**), consistent with a susceptibility phenotype.

Previous EmrE substrate screens have focused on quaternary ammonium compounds (QACs) and quaternary cationic compounds (QCCs) commonly transported by multidrug efflux pumps^1,14–16^. To better explore the substrate profile of EmrE, we performed an unbiased screen using the Phenotypic Microarray assay from Biolog, Inc. This screen assesses the impact of diverse compounds on *E. coli* metabolic output in a differential comparison of MG1655∆*emrE E. coli* expressing either wildtype or nonfunctional EmrE (E14Q-EmrE). If the metabolic output was greater when wild-type EmrE was expressed, it indicates that functional EmrE is beneficial, and the compound was classified as a resistance hit. If the metabolic output was greater when E14Q-EmrE was expressed, it indicates that functional EmrE is detrimental and the compound was classified as a susceptibility hit (Fig. 2, Supplementary Figs. 2, 3, Supplementary Table 1, and see methods for selection criteria). As shown in Fig. 2a, the screen identified compounds in both categories: resistance and susceptibility.

The well-established EmrE substrate methyl viologen (MV^2+^) was the strongest resistance hit with the highest possible score according to our criteria (+8). Acriflavine, another known substrate, was also a strong +4 resistance hit, confirming that the Biolog assay accurately reports on EmrE toxin resistance phenotypes. Chelerythrine chloride has not been previously identified as an EmrE substrate but showed a strong +5 resistance phenotype. Microplate growth assays of *E. coli* expressing either wildtype or E14Q-EmrE in the presence of MV^2+^ or chelerythrine chloride confirmed the resistance phenotype (Fig. 2c, d and Supplementary Fig. 4g–i). Chelerythrine chloride has been used as an antibacterial agent for toxin-resistant infections^17^, so EmrE-conferred resistance may be clinically relevant.

EmrE’s resistance activity has been well characterized in *E. coli*, but a susceptibility phenotype for WT-EmrE has not previously been reported. The top three susceptibility hits identified in the Biolog screen were: harmane (−6), hexachlorophene (−6), and 18-crown-6-ether (−5). Compared to the other susceptibility hits, hexachlorophene is extremely insoluble therefore, it was not evaluated further. In microplate growth assays, E14Q-EmrE expressing cells had a higher final OD_600_ at stationary phase in the presence of 18-crown-6-ether (Fig. 2e, red line and Supplementary Fig. 4d–f) or harmane (Fig. 2f, red line and Supplementary Fig. 4a–c), but cells expressing WT-EmrE had significant growth deficiencies after five hours of treatment (black lines), manifested by an earlier onset of stationary phase with lower OD_600_. This confirmed that functional EmrE confers susceptibility rather than resistance to these compounds. 18-crown-6-ether has previously been implicated in cellular toxicity due to interference with cation transport^18–20^, but the mechanism of the possible antimicrobial activity of harmane is unknown^21,22^.

### Harmane directly binds EmrE

**Fig. 3:**
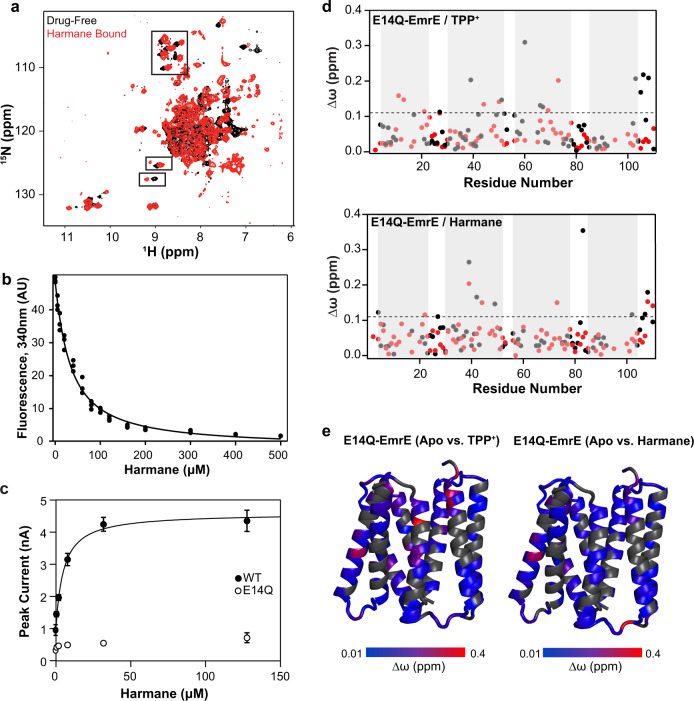
EmrE directly binds harmane in vitro. **a** ^1^H^15^N-TROSY-HSQC spectra of WT-EmrE in isotropic bicelles (*q* = 0.33) at 45 °C without (black) or with (red) harmane bound. Black boxes highlight some of the localized chemical shift perturbations observed upon harmane binding to EmrE. **b** Harmane quenches intrinsic tryptophan fluorescence of WT-EmrE in a dose-dependent manner, with an apparent *K*_d_ value of 29 ± 2 μM. This data (**a**, **b**) confirms that harmane binds WT-EmrE directly at a specific site. **c** SSME measurement of EmrE transport current shows that peak current increases with harmane concentration and saturates, consistent with transport gated by harmane binding. The same 2-fold proton gradient (pH 7.0 outside and pH 7.3 inside) was used as in Fig. 4. The transport fits Michaelis–Menten kinetics with a *K*_m_ value of 5 ± 1 μM. The nonfunctional control (E14Q) displayed no significant current at any concentration of harmane, confirming that harmane-induced proton flux through EmrE requires functional transporter. Data from three independent sensors are presented as the mean value ± SD. **d** Backbone amide chemical shift perturbations of EmrE upon drug-binding (∆ω = ω_drug free_–ω_drug bound_) were measured in isotropic bicelles using NMR. CSPs are shown for binding TPP^+^ (top) or harmane (bottom) to E14Q-EmrE. Data were plotted for chain A (red) and chain B (black) as a function of residue number with the TM helices 1–4 indicated by the gray shading. Residual CSPs at the end of TM2, in the TM3-4 loop, and the C-terminal tail show that EmrE substrates can interact with a secondary binding site in EmrE. **e** Backbone amide CSP data was plotted on the faRM model structure of EmrE^56^. Residues colored in grey are unassigned or overlapped and CSP cannot be calculated. The magnitude of the CSP corresponds to the color bars under each structure.

We acquired ^1^H-^15^N HSQC NMR spectra of WT-EmrE in the presence and absence of harmane (Fig. 3a) to determine whether a direct binding interaction between EmrE and harmane could be responsible for the susceptibility phenotype. The NMR chemical shift perturbations (CSPs) observed for a subset of peaks upon harmane binding indicate that there is the direct interaction between EmrE and harmane at a localized binding site. In the absence of substrate, alternating access of EmrE occurs on the intermediate timescale leading to many broadened peaks (black spectrum). Upon harmane binding, additional peaks are resolved in locations where resolved peaks are observed for EmrE bound to other substrates with experimentally established alternating-access rates in the slow-exchange regime^13^, indicating that harmane binding likely slows the rate of alternating access in EmrE. The extensive dynamics in the drug-free state preclude backbone assignment and residue-specific CSP calculation. We, therefore, used S64V-EmrE for more quantitative CSP analysis of harmane binding, S64V-EmrE is a mutant we recently discovered that slows the internal dynamics of EmrE, dramatically improving the quality and resolution of NMR spectra, but maintains the same binding affinity for TPP^+^ and related substrates^23^. We confirmed that this mutant also binds harmane with comparable affinity to WT-EmrE using intrinsic tryptophan fluorescence quenching^12^ (Fig. 3b and Supplementary Fig. 5). Comparing the NMR CSP induced by TPP^+^ and harmane with S64V-EmrE reveals that both induce CSPs in the middle of transmembrane helix 1 and 3, the regions of EmrE in close proximity to E14. This data shows that both substrates can interact with the known binding site at E14, as visualized in recent monobody-assisted crystal structures of EmrE with each of these ligands^24^. However, the magnitude and extent of the CSPs are strikingly different for these two substrates. TPP+ induces large CSPs throughout much of the transporter, indicating that TPP^+^ binding induces large-scale conformational changes in EmrE. In contrast, harmane shows much more localized CSPs consistent with harmane binding without causing significant structural change.

Twenty years ago, ^31^P solid-state NMR studies of TPP^+^ binding to EmrE showed the presence of a second drug-binding site in the loops between the TM helices^25^. Those experiments suggested that TPP^+^ bound first to this peripheral low-affinity site before binding to the high-affinity site defined by E14. To probe whether harmane might interact with this putative ill-defined secondary site, we mutated E14 to eliminate the primary drug-binding site and performed NMR CSP studies of harmane and TPP^+^ binding with E14Q-EmrE (Fig. 3d, e). With the well-established primary drug-binding site abolished^3,26,27^, any CSPs observed upon addition of the drug must be due to interactions with secondary binding sites in EmrE. Indeed, small but statistically significant CSPs are observed for both TPP^+^ (top panel, Fig. 3d) and harmane (bottom panel, Fig. 3d) binding to E14Q-EmrE. Unlike S64V-EmrE (Supplementary Fig. 6a, b, e, f), where harmane and TPP^+^ have very different CSP profiles, the magnitude and pattern of CSPs is comparable for either substrate binding to E14Q-EmrE, with CSPs located in the C-terminal tail, the C-terminal half of TM2, and the TM3-TM4 loop (Fig. 3d, e and Supplementary Fig. 6c, d, g, h). Due to the antiparallel topology of the EmrE homodimer, the 2–3 loop and C-terminal tail of one protomer are near the 3–4 loop from the other protomer. TM2 has been shown to be involved in substrate specificity^16^, and the loops and tail have previously been suggested to be involved in a secondary binding site and participate in drug- and proton-binding^25,28,29^. These results are consistent with a putative secondary binding site in this region of EmrE. While TPP^+^ also interacts with this peripheral site, TPP^+^ interactions with the primary binding site are much stronger^25,30^, such that interactions with the peripheral site are only apparent when the primary E14 binding site is removed by mutation. This suggests not only that both drugs can interact with EmrE in the absence of the primary binding site E14, but that the mode of this secondary interaction is potentially similar.

### Harmane triggers uncoupled proton transport

To determine whether harmane binding triggers EmrE transport activity, we turned to solid-supported membrane electrophysiology (SSME). SSME allows the detection of net charge movement in proteoliposomes adsorbed onto a gold electrode sensor upon buffer perfusion and is ideal for measuring small transport currents produced by moderate-flux transporters such as EmrE^31^. Harmane triggered EmrE transport currents, which increased and eventually saturated with increasing harmane concentrations (Fig. 3c). While this data is strongly suggestive of a direct effect of harmane on the transport activity of EmrE, how might EmrE transport lead to drug susceptibility?

EmrE-mediated drug resistance phenotypes can only be explained by the canonical proton/drug antiport mechanism, but toxin susceptibility can arise from three potential transport mechanisms: drug uniport, proton uniport, or proton/drug symport (Fig. 1). To better understand how EmrE confers susceptibility to harmane, we performed additional SSME experiments using an assay recently developed in our lab to characterize the ion-coupling behavior of secondary active transporters^32,33^.

**Fig. 4:**
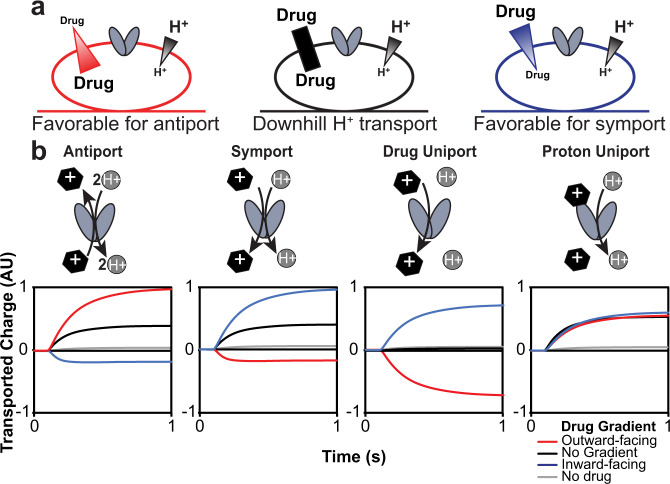
Determination of transport mode in vitro using solid-supported membrane electrophysiology (SSME). Expected results in the SSME assay for the different transport modes of EmrE as predicted by the Free Exchange Model. **a** Three conditions are used to determine the transport mode of EmrE using SSME. In all cases, there is a twofold inward-driven proton gradient (pH 7.0 outside and pH 7.3 inside). To test for antiport (red), a 16-fold substrate gradient was pointed against the proton gradient. For symport (blue), the 16-fold drug gradient is oriented with the proton gradient. A third condition testing the presence of a drug without a gradient (black) acts as a control for drug-activated proton uniport. **b** Cartoon models of the expected behavior under each condition are shown for different defined modes of transport of a +1 substrate. The cartoons highlight which substrates cross the membrane, as that defines the net charge movement monitored in SSME. Note that Proton Uniport denotes drug-gated proton uniport, not uncontrolled proton leak by the transporter alone. For more detailed models of expected behavior with both positively charged and neutral substrates, please see Supplementary Fig. 7.

The hallmark of coupled transport is the ability of the downhill transport of one substrate to drive the uphill transport of another substrate. The difference between antiport and symport is simply which orientation of the drug gradient (relative to the smaller proton gradient) enhances proton-driven transport and which orientation reverses net transport. In the SSME assay, transport is initiated by buffer perfusion to create substrate gradients across the liposomal membranes. Various combinations of substrate gradients (Fig. 4a) will have different and predictable effects on the transport signal in the case of antiport, symport, drug uniport, or proton uniport (Fig. 4b).

In the absence of a drug gradient (black), transport is driven by the twofold inward-facing proton gradient, resulting in a positive signal for the canonical 2 H^+^/1 drug^+^ antiport (net +1 inward per transport cycle), symport (net +1 or +2 inward, for H^+^ and a neutral or drug^+^), or proton uniport (net +1 inward). For drug uniport, a proton gradient alone will not drive transport, resulting in no signal. In the case of drug/proton antiport, the addition of a much larger drug gradient opposite the proton gradient (red) will favor the antiport and cause a larger positive signal, while aligning the drug- and proton-gradients in the same direction (blue) requires one substrate to move against its concentration gradient. Under our experimental conditions, the driving force from the drug gradient out-competes the proton gradient and reverses the direction of net transport compared to the twofold proton gradient alone. In contrast, uncoupled transport depends solely on the gradient of the uniported substrate. Drug uniport depends only on the direction of the imposed drug gradient and the net charge on the drug (shown for drug^+^, no current would be observed under any condition for an uncharged substrate such as harmane). Proton uniport will result in a consistent, positive signal due to proton flux down the uniform twofold proton gradient under all three conditions. Transport should be minimal in the absence of the drug (gray) as EmrE does not spontaneously leak protons^4^. A detailed explanation of the SSME assay is available in the Supplementary Data along with an expanded diagram of the expected results for both positively charged and neutral substrates (Supplementary Fig. 7). To have well-defined substrate and proton gradients, and thus a well-defined driving force for transport, both proton and substrate must be present on both sides of the membrane at known concentrations. Thus, liposomes are pre-loaded with a specific concentration of each substrate in a buffer at a specific pH. The new external buffer is then flowed in with substrate and pH set to create the precise gradient (or no-gradient) conditions.

**Fig. 5:**
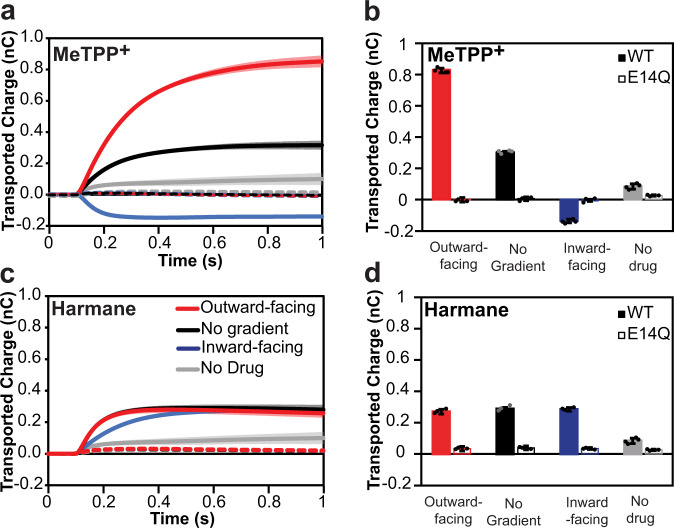
Harmane induces proton flux through EmrE. MeTPP^+^ (**a**, **b**) behaves as expected for an antiported substrate (see Fig. 3). The total transported charge reverses when the drug gradient is inverted, characteristic of coupled antiport. In contrast, the harmane transport signal (**c**, **d**) is the same regardless of the harmane gradient, matching the expected behavior for downhill proton transport (proton leak). The current is minimal in the absence of drug (**b**, **c**; gray) or for liposomes containing nonfunctional E14Q-EmrE (dashed lines), indicating that the observed charge transport is due to substrate-triggered EmrE activity. Data are presented as the mean value ± SD. Wild-type EmrE data were obtained from four independent sensors. E14Q-EmrE data were obtained from three independent sensors. These values are indicated on the corresponding bar graphs.

We first measured net charge movement under the three gradient conditions depicted in Fig. 4a for the transport of methyl tetraphenylphosphonium (MeTPP^+^), which is known to be antiported by EmrE (Fig. 5a, b and Supplementary Fig. 8a, b). Proteoliposomes reconstituted with E14Q-EmrE (dashed lines) were used as negative controls and produced minimal signals under all conditions, regardless of substrate. In the absence of the drug, the proton gradient alone induces a small positive current in WT-EmrE proteoliposomes, indicating minimal proton leak. When MeTPP^+^ is added, we observe transport reversal as expected for 2 H^+^/1 MeTPP^+^ antiport.

In contrast, net transport does not reverse when harmane is the substrate (Fig. 5c, d and Supplementary Fig. 8c, d). Instead, the net charge transport is constant and positive (down the proton gradient), under all conditions. It is important to note that harmane is potentially uncharged under these assay conditions. Thus, any net charge movement is actually the movement of protons (H+). However, the assay can still distinguish coupled and uncoupled transport because the driving force provided by the harmane gradient will affect the direction of proton movement if there is coupled transport of harmane and proton(s). This lack of reversal of net charge movement between inwardly and outwardly directed drug gradients is indicative of uncoupled proton transport (leak). The fact that the system reaches a steady state at the same total net charge movement under all three conditions indicates that the same thermodynamic driving force is driving transport in each case. In our assay setup, the proton gradient is constant while the drug gradient is varied, so this means that transport is driven solely by the proton concentration gradient and must reflect proton leak, with no coupled harmane transport. However, this uncoupled proton leak is triggered by (or gated by) harmane since the signal is significantly larger with harmane than the background proton leak in the absence of the drug and is dependent on harmane concentration, as discussed below. These assay results also exclude dissipation of ∆pH by harmane diffusion through the membrane in an EmrE-independent manner, as explained in more detail in Supplementary Fig. 8. Thus, EmrE can function as a harmane-gated proton transporter in addition to its well-established proton-coupled antiport of a broad class of polyaromatic cation substrates.

### Harmane dissipates the ∆pH component of the proton motive force

Reexamining the cell growth assays indicates the significant harmane phenotype appears around the 5-h mark (Fig. 2e, f), approximately the point at which fermentable sugars are consumed^34^ and cells become more reliant on the proton motive force for ATP production when *E.coli* are grown in LB. Thus, the in vitro and in vivo assays so far are consistent with harmane triggering an EmrE-mediated uncontrolled proton leak. To explore the mechanism of proton leak in bacteria, we performed checkerboard assays with kanamycin and tetracycline and measured growth curves and IC_50_ values with co-treatment of harmane and bicarbonate. These assays are indirect, but are commonly used to test whether small molecules dissipate the ∆pH in bacteria^35,36^.

**Fig. 6:**
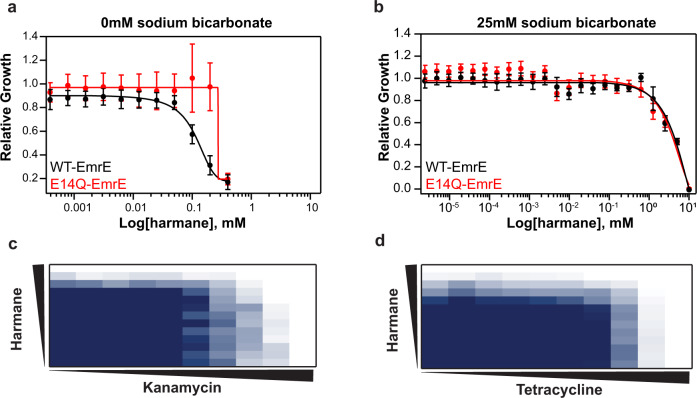
Harmane dissipates the proton gradient of bacterial cells. **a**, **b** Relative growth was plotted as a function of harmane concentration in the presence of 25 mM sodium bicarbonate (**b**) for MG1655 ∆*emrE E. coli* cells expressing WT- (black) and nonfunctional, E14Q-EmrE (red). Compared to the results of IC_50_ assays without bicarbonate (**a**), bicarbonate diminishes the susceptibility phenotype in *E. coli* expressing WT-transporter. Error from the triplicate measurements is displayed as the standard deviation. MG1655 ∆*emrE E. coli* expressing WT-EmrE were grown in the presence of harmane as an adjuvant with kanamycin (**c**) and tetracycline (**d**). Growth is shown as a gradient of the highest OD_600_ (navy) to no growth (white). The results shown are the average of three biological replicates. In the presence of harmane, it takes less kanamycin to reach the 10% growth mark, as indicated by the step pattern in **c**. This pattern is not seen when harmane is added as an adjuvant to tetracycline. Further, the mean FIC values of the experiments are 0.375 (synergistic), and 0.58 (indifferent) for kanamycin and tetracycline, respectively. Compound concentrations are denoted by the black triangles increasing left to right for kanamycin and tetracycline and bottom to top for harmane.

Bicarbonate itself acts to rapidly dissipate the ∆pH component of the proton motive force by direct diffusion through the membrane in the neutral form, effectively acting as a proton carrier^35^. Consequently, we expect that the harmane phenotype would be reduced in the presence of bicarbonate since if ∆pH has already been dissipated by bicarbonate, the slower process of harmane-gating EmrE to allow proton leak will not have any additional effect. We therefore determined the IC_50_ of MG1655 ∆*emrE* cells expressing WT- or nonfunctional EmrE treated with harmane and bicarbonate simultaneously. In the presence of harmane and bicarbonate (Fig. 6b), there is no phenotypic difference between *E. coli* expressing WT-EmrE (black) and E14Q-EmrE (red). Compared to the IC_50_ curve without bicarbonate (Fig. 6a), the cells are also able to withstand much higher concentrations of harmane in the media. The antagonism between bicarbonate and harmane is consistent with harmane dissipating the ∆pH component of the proton motive. In addition, the differential effect of harmane on cells expressing WT versus nonfunctional EmrE is clear in Fig. 2b, reflecting that harmane acts via EmrE. However, when bicarbonate is added and dissipates the ∆pH in an EmrE-independent manner, there is no longer a significant difference between functional versus nonfunctional EmrE. These results support the in vitro assays indicating harmane triggers uncoupled proton leak through EmrE and dissipates the ∆pH component of the proton motive force through EmrE in vivo.

To determine whether harmane could act as an antibiotic adjuvant, we performed checkerboard assays with kanamycin (Fig. 6c) and tetracycline (Fig. 6d). Checkerboard assays are useful for assessing the interaction between two drug compounds^36,37^. In this assay, two common antibiotics were used, kanamycin and tetracycline. These antibiotics were selected as they have been used in checkerboards previously when studying compounds that may affect the proton motive force. Since harmane appears to be antagonized by sodium bicarbonate, we anticipated that there would be some interaction between harmane and these antibiotics. The mean fractional inhibitory concentration (FIC) index was calculated to determine synergism (FIC < 0.5), indifference (0.5 ≤ FIC < 1), or antagonism (FIC ≥ 1).

Figure 6 shows the results of checkerboard assays with harmane and kanamycin or tetracycline. These assays were performed in MG1655 ∆*emrE E. coli* expressing WT- or nonfunctional EmrE to confirm that harmane-triggered EmrE activity in vivo is due to ∆pH dissipation. In the harmane/kanamycin experiment (Fig. 6c), a characteristic stair pattern is observed as expected for a synergistic relationship between the two compounds. An FIC value of 0.37 was determined, similar to the previous reports^36^ for kanamycin and harmane, suggesting synergy. When the same analysis was performed with tetracycline (Fig. 6d), an FIC value of 0.58 was determined, indicating indifference between tetracycline and harmane. No synergy is observed when *E. coli* expressing nonfunctional transporter are used (Supplementary Fig. 9). In addition, these results demonstrate that small molecules like harmane that trigger proton leak through EmrE may synergize with certain classes of antibiotics with independent modes of action.

## Discussion

SMR transporters are found throughout the bacterial kingdom and efflux toxic hydrophobic cations through coupled antiport of substrate and protons, as illustrated in Fig. 1^38–41^. The most widely studied member of this family, EmrE, confers resistance to a broad array of toxic polyaromatic cations in *E. coli*^16,42^. Although this small protein was originally proposed to be an ideal model for studying the molecular mechanism of proton-coupled antiport, detailed biophysical studies have instead revealed surprising promiscuity not just in substrate specificity, but also in the transport mechanism. The data presented here confirm that alternative transport modes suggested by that prior work can be activated in vivo by small molecules, such that some small molecules trigger EmrE-mediated susceptibility rather than resistance. Our in vitro SSME assay designed to distinguish between different modes of proton-coupled and uncoupled transport clearly shows that the dominant mode of transport by EmrE differs for MeTPP^+^ and harmane. EmrE performs 2 H^+^:1 MeTPP^+^ coupled antiport, as expected for this previously known EmrE substrate. In contrast, harmane binds to EmrE and triggers an uncoupled proton leak, revealing a substrate-gated proton uniport mode for this transporter. The NMR CSP profiles reveal a shared ability of both substrates to bind EmrE in the absence of the primary binding site defined by E14, consistent with a decades-old hypothesis of a secondary binding site for drugs near the surface of the transporter. However, there are distinct differences in the CSP profiles when the E14 binding site is intact. The substrate that is actively transported, TPP^+^, causes widespread CSPs indicative of broad structural change throughout EmrE upon TPP^+^ binding, while the substrate that gates the transporter but is not itself transported causes much smaller and more localized CSPs. Protons are quite small, so opening the transporter sufficiently to allow proton leak would require much less structural rearrangement than would be needed to permit the drug substrate to enter and exit the transport pore. Additional large-scale screening will be needed to better define the chemical space of EmrE substrates and identify the physicochemical features of the small molecule and specific small molecule-protein interactions needed to trigger uncoupled proton leak rather than proton-coupled toxin efflux.

Uncontrolled proton leak through EmrE will dissipate the transmembrane pH component of the proton motive force in vivo, consistent with time-dependent growth defect (Fig. 2e, f) and antagonism between harmane and bicarbonate (Fig. 6b). Targeting bacterial bioenergetics as an alternative to cell envelope biogenesis or macromolecular biosynthesis is an area of active interest for antibiotic development^36,37,43,44^ as well as synergy or collateral susceptibility with current antibiotics^45–48^. While several PMF-targeting molecules are available such as gramicidin or nigericin, they generally act non-specifically by creating pores in the lipid bilayer. In other cases, such as the recently identified antimicrobial halicin, the PMF is targeted through an unknown mechanism^37^. Interestingly, harmane itself has previously been observed to prolong the life of *C. elegans* in the presence of different bacteria through an unknown mechanism^21^, and our results suggest one possible reason for that result. The results presented in this paper demonstrate that small molecules can trigger alternative transport modes in a small multidrug resistance transporter, including ligand-gated proton flux. Furthermore, the in vivo data demonstrate that the proton leak triggered by harmane is significant enough to be detrimental to cell growth and metabolism and can synergize with the activity of existing antibiotics that utilize other components of the proton motive force.

Kinetic studies of purified transporters show that some transporters appear to be tightly coupled and highly efficient while others are more loosely coupled, although the experimental challenges of performing these experiments have limited the number of transporters whose mechanism has been rigorously characterized. More recently, there has been renewed interest in kinetic modeling to understand how these more complex network models still achieve relatively efficient coupled transport and may be important for optimizing overall biological function^11,49,50^. In ATP-coupled transport systems, a more significant “leak” (uncoupled ATP hydrolysis) is observed for promiscuous transporters than for highly selective transporters. For example, the multidrug efflux pump P-glycoprotein exhibits significant levels of basal ATP hydrolysis^51^. Loose coupling between the driving force, whether that consists of an electrochemical ion gradient or ATP hydrolysis, and substrate transport may be a requirement of multidrug recognition and efflux, as tight binding generally requires highly specific and selective interactions between the protein and the substrate. The possibility that loose coupling would extend to ion-coupled multidrug transporters, including the SMR family, was originally discussed more than 20 years ago^52^. Here we show that a small molecule can exploit this property of promiscuous multidrug transporters and trigger protein-mediated proton leak. If the loose coupling is required for multidrug efflux, targeting dissipative pathways in multidrug transporters may represent a general strategy for combatting antibiotic resistance, either through the development of proton motive force-dissipating antibiotics or in combination to restore the efficacy of current antibiotics.

## Methods

### Plasmids and strains

All in vivo experiments were performed in MG1655 *∆emrE E. coli* cells (Item number: JW0531-2, *E. coli* Genetic Resource (CGSC), Yale) transformed with a low copy number plasmid under a pTrc promoter. In vivo experiments relied on leaky expression of these plasmids and expression levels were validated by western blot analysis (Fig. S1). Protein expression utilized BL21 (Gold) DE3 *E. coli* transformed with a pET15b plasmid containing the respective EmrE construct. Detailed validation of the pWB plasmid expression, as well as vector controls, can be found in Fig. S1. Expression levels were validated from the pWB plasmid by western blot using an anti-His HRP conjugate kit (Qiagen) (Supplementary Fig. 1).

### Biolog phenotypic microarrays

MG1655 *∆emrE E. coli* cells expressing either WT- or E14Q-EmrE constructs were grown on lysogeny broth media with ampicillin overnight at 37 °C. The phenotype microarray tests followed the established protocols of standard phenotype microarray (PM) procedures for *E. coli* and other gram-negative bacteria^53^. PM1120 plates were used to screen both WT- and E14Q-EmrE expressing *E. coli* [https://www.biolog.com/]. Overnight plates were resuspended in IF-0a inoculating fluid (Biolog) to an optical density of 0.37. Cells were diluted to a 1:200 dilution in IF-10a media (Biolog) with Redox Dye A for PM9-20 plates. PM plates were inoculated with 100 µL of cell suspensions per well. The microplates were incubated at 37 °C and read using the OmniLog instrument every 15 min for 24 h. The area under the resulting metabolic curves was determined for cells expressing WT-EmrE or E14Q-EmrE. The difference was calculated using the equation:

1 $$\triangle {{{{\rm{Area}}}}}={{{{{\rm{Area}}}}}}_{{{{{\rm{WT}}}}}}-{{{\mbox{Area}}}}_{{{{{\rm{E}}}}}14{{{{\rm{Q}}}}}}$$

This equation resulted in positive values for greater respiration by cells expressing WT-EmrE and negative values for greater respiration by cells expressing nonfunctional EmrE. This Delta value was calculated for each replicate separately (WT1-E14Q1, WT2-E14Q2) and the 10% trimmed mean of Delta value was calculated for each of the two replicates. The replicates were handled separately because variation between replicates can arise due to minor deviations between plate sets or in the exact concentration of dye or OD of cells upon dilution on different days. The standard deviation was then calculated among known non-hits (selecting at least 50 wells out of the 960 total wells in a single data set) to determine the cut-off values for actual hits. Individual wells were assessed as hits if the calculated Delta value (Eq. 1) was more than two standard deviations from the 10% trimmed mean. For each hit, a value of +1 was assigned for resistance hits (positive Delta), and a value of −1 was assigned for susceptibility hits (negative Delta). These values were then summed across all eight wells for a single compound (four wells of the same compound per plate set × 2 replicates, with a max score of ±8. Final resistance or susceptibility hits were assigned if the total score was ≥+3 (resistance) or ≤−3 (susceptibility). This definition was chosen since small total hit scores of ±1 or ±2 could arise by chance using the ±2 × SD cutoff to score individual wells. Values of ±3 recognize consistent hits across multiple replicates and/or different concentrations of the same compound. Our cutoff is not set higher since the four wells of each compound on a single plate set include different concentrations and some concentrations may not be sufficient to elicit a phenotype.

### Microplate growth assays

Cells expressing plasmids of interest were grown in Mueller-Hinton broth (Sigma, 100 µg/mL ampicillin, pH 7.0) from a single colony to an OD of 0.2 at 37 °C. The cells were then diluted to a final OD of 0.01 in 384-well microplates containing concentration ranges of MV^2+^, harmane, 18-crown-6-ether, and chelerythrine chloride. The plates were incubated and shaken in a microplate reader (BMG-Labtech) at 37 °C. OD_600_ was measured every 5 min for 20 h. Experiments were performed with four biological replicates and data were analyzed using Igor Pro v7.0.8.1 (WaveMetrics Inc.).

### IC_50_ value determination

MG1655 *∆emrE E. coli* cells expressing either WT or E14Q-EmrE were grown overnight at 37 °C from a single colony. Concentration ranges of ethidium bromide (0–5 mM) and harmane (0–0.4 mM) were assayed in microplates with a starting OD_600_ of 0.1. Plates were then incubated with shaking for 18 h with shaking at 37 °C. OD_600_ endpoints were taken using a BMG plate reader. Relative growth was calculated by dividing the measured OD_600_ from a given concentration by the OD_600_ for cells containing no drugs. Experiments were performed in triplicate and fit a simple sigmoid equation using Igor Pro v7.0.8.1 (WaveMetrics Inc.).

### EmrE expression and purification

BL21 Gold (DE3) *E. coli* cells were transformed with pET15b-WT-EmrE, pET15b-S64V-EmrE, or pET15b-E14Q-EmrE plasmids and grown in M9 minimal media to an OD_600_ of 0.9. The bacteria were flash-cooled and then induced with 0.33 M IPTG overnight at 17 °C. The *E. coli* cells were collected with centrifugation, lysed, the membrane fraction solubilized with decylmaltoside (DM), and the proteins purified using nickel affinity chromatography followed by size exclusion chromatography (SEC) on a Superdex 200 column as previously described in ref. 13. Protein concentrations were determined using absorbance at 280 nm with an extinction coefficient of 38,400 L/mol cm^54^.

### Intrinsic tryptophan assays

Purified WT- and S64V-EmrE were reconstituted into isotropic bicelles of DMPC/DPHC (*q* = 0.33) as previously described in ref. 55. Reconstitution of purified EmrE into liposomes was performed as described above for SSME transport assays but using DMPC lipids with an EmrE:DMPC ratio of 1:75. Bicelle stocks (2X) were prepared by dissolving DMPC in assay buffer containing 100 mM MOPS pH 7.0, 20 mM NaCl to a final concentration of 300 mM and incubating at 45 °C for 1.5 h. DHPC was then added to a final concentration of 100 mM to create *q* = 0.33 isotropic bicelles, incubated for an additional hour, and subjected to three freeze/thaw cycles. Harmane was prepared to a maximal concentration of 800 μM in assay buffer with 1X bicelle stock and rotated for 72 h, then serial diluted into black 96-well flat-bottom plates. WT- and S64V-EmrE in DMPC/DHPC bicelles were added to a final dimer concentration of 10 μM and the plate was incubated at room temperature for 1 h. The final assay volume was 200 μL, and each concentration was present in triplicate. Endpoint fluorescence was determined using a TECAN Spark and data analysis was performed in Igor Pro v8. The emission wavelength was 280 nm (15 nm bandwidth) and the excitation wavelength was 340 nm (20 nm bandwidth) and the measurement integration time was 40 μsec with 50 flashes. The Z-position and gain were determined automatically by the TECAN instrument from the A1 position.

Data were fit to a single binding isotherm detailed in the following equation:

2 $${F}_{{{{{\rm{obs}}}}}}={F}_{{{{{\rm{EH}}}}}}\left[{{{{\rm{EH}}}}}\right]+{F}_{{{{{\rm{E}}}}}}[{{{{\rm{E}}}}}]$$

Where *F*_obs_ is the observed fluorescence, *F*_EH_ is the fluorescence of the EmrE functional dimer bound to harmane, [EH] is the concentration of EmrE functional dimer bound to harmane, *F*_E_ is the fluorescence of the EmrE functional dimer, and [E] is the concentration of EmrE functional dimer.

[EH] is calculated from the following equation:

3 $$\left[{{{{\rm{EH}}}}}\right]=\frac{\left({{{{{\rm{E}}}}}}_{{{{{\rm{T}}}}}}+{{{{{\rm{H}}}}}}_{{{{{\rm{add}}}}}}+{{{{{\rm{K}}}}}}_{{{{{\rm{d}}}}}}\right)-\sqrt{{\left({{{{{\rm{E}}}}}}_{{{{{\rm{T}}}}}}+{{{{{\rm{H}}}}}}_{{{{{\rm{add}}}}}}+{{{{{\rm{K}}}}}}_{{{{{\rm{d}}}}}}\right)}^{2}-4{{{{{\rm{E}}}}}}_{{{{{\rm{T}}}}}}{{{{{\rm{H}}}}}}_{{{{{\rm{add}}}}}}}}{2}$$

Where E_T_ is the total concentration of EmrE functional dimer in the sample, H_add_ is the total added harmane in the sample, and *K*_d_ is the dissociation constant.

The concentration of unbound EmrE functional dimer ([E]) is given by the following equation:

4 $$\left[{{\mbox{E}}}\right]=1-[{{\mbox{EH}}}]$$

### Direct binding by NMR spectroscopy

Purified ^15^N^2^H-EmrE (0.7–1 mM) was reconstituted into isotropic bicelles (*q* = 0.33) at pH 4.5. The harmane-bound EmrE sample was soaked in harmane overnight with incubation at 45 °C. HN-Transverse relaxation optimized spectroscopy—heteronuclear single quantum correlation (HN-TROSY-HSQC) experiments were performed on a 750 MHz Bruker Avance spectrometer at 45 °C (d1 = 2 s). Spectra were processed and analyzed using NMRPipe and CCPnmr Analysis 3.0.4.

### Chemical shift perturbation mapping

Purified ^15^N^2^H-S64V-EmrE and ^15^N^2^H-E14Q-EmrE (0.7–1 mM monomer concentration) were reconstituted into isotropic bicelles (*q* = 0.33) at pH 5.6. The harmane-bound EmrE sample was soaked in harmane overnight with incubation at 45 °C. TPP^+^-bound samples were saturated with 16 mM TPP^+^. HN-Transverse relaxation optimized spectroscopy—heteronuclear single quantum correlation (HN-TROSY-HSQC) experiments were performed on a 750 MHz Bruker Avance spectrometer at 45 °C (d1 = 2 s). Backbone assignment experiments (3D HNCO, 3D HNCA, 3D HNcoCA, 3D HNcaCO) were performed at 750 and 900 MHz using Bruker TopSpin software with the unbound, TPP^+^-bound, and harmane-bound samples at 45 °C. Additional experimental details can be found in the BMRB and BMRbig depositions for these spectra. Spectra were processed and analyzed using NMRPipe and CCPnmr Analysis 3.0.4.

### Solid-supported membrane electrophysiology transport assays

WT- and E14Q-EmrE were expressed and purified, with the final SEC performed in assay buffer (50 mM MES, 50 mM MOPS, 50 mM bicine, 100 mM NaCl, 2 mM MgCl_2_, 40 mM DM, pH 7). All buffers were carefully adjusted to the desired pH exclusively with NaOH to ensure consistent Cl^-^ concentrations across the membrane for transport assays. Protein was reconstituted into 1-palmitoyl-2-oleoyl-*sn*-glycero-3-phosphocholine (POPC) proteoliposomes at a lipid-to-protein ratio of 1:400 in pH 7 assay buffer. Briefly, 15 mg/ml stocks of POPC were diluted in assay buffer and incubated at 45 °C for 1 h. Lipids were bath sonicated for 1 min then octyl glucoside (OG) was added to a final concentration of 0.5%. Lipids were sonicated for an additional 30 seconds and returned to 45 °C to incubate for 15 min. SEC fractions containing purified protein in DM were added to the lipid solution and incubated at RT for 25 min, then detergent was removed with Amberlite XAD-2 as previously described in ref. 55. As a negative control, POPC lipids were put through a simulated reconstitution process without protein. Amberlite was removed from each sample via a gravity column and uniform liposomes were obtained by extrusion through a 0.2 µM membrane using an Avanti MiniExtruder. All SSME data were acquired using a Nanion SURFE2R N1 instrument. Liposome aliquots were thawed, diluted twofold, and briefly sonicated. About 10 μL of liposomes were used to prepare 3 mm sensors as previously described in ref. 33. Before experiments, sensor capacitance and conductance values were obtained to ensure sensor quality. Based on manufacturer recommendations, sensors used were limited to those with a capacitance of 15–35 nF and a conductance <5 nS. However, capacitance ranges were generally closer to ~20 nF and conductance of 1–2 nS. Before experiments, sensor capacitance and conductance values were obtained to ensure sensor quality. For all experiments, buffers contained 50 mM MES, 50 mM MOPS, 50 mM bicine, 100 mM NaCl, and 2 mM MgCl2 with internal pH values of 7.3 and external pH values of 7.0. For inward-facing drug gradients, external drug concentration was 8 μM and internal drug concentration was 0.5 μM. For outward-facing drug gradients, internal drug concentration was 8 μM and external drug concentration was 0.5 μM. Both internal and external drug concentration was 8 μM for the zero-gradient data. Sensors were rinsed with at least 500 μL of internal buffer before each measurement to set the internal buffer, pH, and drug concentrations as described in^33^. Measurements were performed at a flow rate of 200 μL sec^−1^. Data acquisition occurred in three stages. First, sensors were perfused with an internal buffer, then transport was initiated by perfusion of the external buffer, and finally, perfusion of the internal buffer re-equilibrated the sensors. Signals were obtained by integrating the current during the perfusion of the external buffer, with the final 100 ms of the initial internal buffer perfusion used as the baseline. Reported data are average values of at least three sensors, with error bars representing the standard error of the mean.

### Checkerboard assays

MG1655 *∆emrE E. coli* cells expressing either WT- or E14Q-EmrE constructs were grown in Mueller-Hinton broth (Sigma, 100 µg/mL carbenicillin, pH 7.0) from a single colony to an OD of 0.2 at 37 °C. Kanamycin or tetracycline was serially diluted across a 96-well microplate in MHB with concentrations ranging from 0–80 μM or 0–16 μM, respectively. Harmane was serially diluted down a separate plate using MHB with concentrations ranging from 0–1150 μM. The cells were then diluted to a final OD of 0.01 in the microplate. A column with no kanamycin or tetracycline and a row with no harmane was used to determine the MIC values for each compound. Inoculated plates were sealed and incubated with shaking for 18 h at 37 °C. OD_600_ endpoints were taken using a microplate reader (BMG-Labtech). Checkerboard synergy testing was performed in triplicate and analyzed for MIC and FIC values in Excel.

The fractional inhibitory concentration (FIC) index was calculated for each well with no turbidity along the interface using the MIC values for the different compounds individually and in tandem. The MIC value was defined as the minimum concentration required to inhibit all cell growth to 10% of the background growth, as detailed in ref. 36. FIC values were determined using the following equations:

5 $${{{{{{\rm{FIC}}}}}}}_{({{{{{\rm{A}}}}}})}=\frac{{{{{{{{\mathrm{MIC}}}}}}}}_{(A+B)}}{{{{{{{{\mathrm{MIC}}}}}}}}_{(A)}}$$

6 $${{{{{{\rm{FIC}}}}}}}_{({{{{{\rm{B}}}}}})}=\frac{{{{{{{{\mathrm{MIC}}}}}}}}_{(B+A)}}{{{{{{{{\mathrm{MIC}}}}}}}}_{(B)}}$$

7 $${{{{{\rm{FIC}}}}}}={{{{{{\rm{FIC}}}}}}}_{({{{{{\rm{A}}}}}})}+{{{{{{\rm{FIC}}}}}}}_{({{{{{\rm{B}}}}}})}$$

where A and B represent the different compounds in the assay. The mean FIC index was calculated and used to determine synergism (FIC < 0.5), indifference (0.5 ≤ FIC < 1), or antagonism (FIC ≥ 1).

### Bicarbonate assays

MG1655 *∆emrE E. coli* cells expressing either WT or E14Q-EmrE were grown overnight at 37 °C from a single colony. Harmane was serially diluted across a 96-well microplate in Mueller-Hinton broth (Sigma, 100 µg/mL ampicillin, pH 7.0) from 0–0.4 mM, with or without 25 mM bicarbonate (pH 7.4), and assayed with a starting OD_600_ of 0.01. Plates were then sealed and incubated with shaking for 18 h at 37 °C. OD_600_ endpoints were taken using a microplate reader (BMG-Labtech). Relative growth was calculated by dividing the measured OD_600_ from a given concentration by the OD_600_ for cells containing no drug. IC_50_ curves were performed in triplicate with three biological replicates per plate. Data were fit to a simple sigmoid equation using Igor Pro v8 (WaveMetrics Inc.).

### Reporting summary

Further information on research design is available in the Nature Portfolio Reporting Summary linked to this article.

## Supplementary information

Supplementary Information

Reporting Summary

## Data Availability

The source data and analyzed datasets generated in this study are available in the MendeleyData repository [10.17632/tpcdgw7h6m.1]. NMR chemical shift data can be found on the Biological Magnetic Resonance Data Bank (BMRB) under accession numbers 51024 (S64V-EmrE, drug-free, pH 5.0), 51025 (S64V-EmrE with harmane, pH 5.6), 51026 (S64V-EmrE with TPP, pH 5), 51027 (E14Q-EmrE drug-free, low pH), 51030 (E14Q-EmrE with 16 mM TPP, low pH), and 51031 (E14Q-EmrE with harmane, low pH). Raw NMR data were deposited on the BMRbig database [https://bmrbig.bmrb.io/] under accession numbers BMRbig18, BMRbig19, BMRbig20, BMRbig21, BMRbig22, and BMRbig23. Uncropped western blots are supplied in the Supplementary Information.

## Code availability

The relevant scripts to generate plots and handle data were matched with their data types in the MendeleyData repository [10.17632/tpcdgw7h6m.1].
