# Supplementary material for: Activating alternative transport modes in a multidrug resistance efflux pump to confer chemical susceptibility
Source: Nat Commun. 2022 Dec 10;13:7655. doi: 10.1038/s41467-022-35410-2 (PMC9741644; doi:10.1038/s41467-022-35410-2)

Supplementary Information

Activating alternative transport modes in a multidrug resistance efflux pump to confer chemical susceptibility

Peyton J. Spreacker[1,3], Nathan E. Thomas[1,3]#, Will F. Beeninga[1]§, Merissa Brousseau[1], Colin J. Porter[1], Kylie M. Hibbs[1], Katherine A. Henzler-Wildman[1,2]*

*Corresponding Author: Katherine A. Henzler-Wildman
Email: henzlerwildm@wisc.edu

This PDF file includes:

Supplementary Fig. 1 - 8
Supplementary Tables 1, 2
Uncropped blots from Supplementary Fig. 1

Other supplementary materials for this manuscript include the following:

Biolog microarray data, SSME raw data/analysis, IC_{50} replicate data, Intrinsic Tryptophan Fluorescence data, and heatmap scripts (https://doi.org/10.17632/tpcdgw7h6m.1)

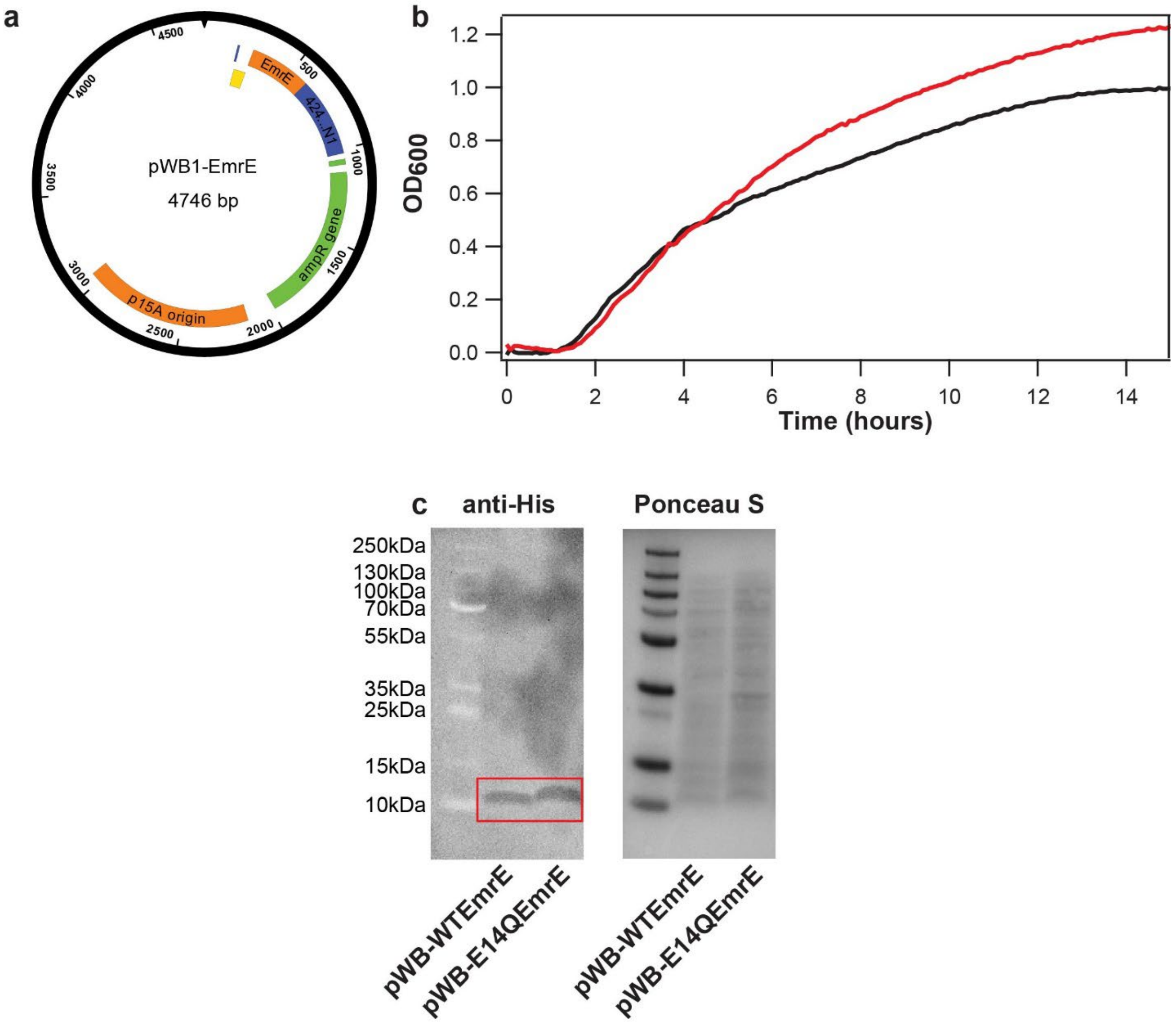

Supplementary Figure 1: Leaky expression of WT-EmrE and non-functional, E14Q-EmrE have equal expression levels and similar growth patterns. (a) The pWB plasmid expresses EmrE (WT or E14Q) under the control of a pTrc promoter. All *in vivo* experiments were carried out using only leaky expression of EmrE without induction by IPTG. (b) MG1655 Δ*emrE* cells expressing WT- or E14Q-EmrE were grown in Mueller-Hinton Broth with ampicillin in the absence of drug treatment. Note that E14Q-EmrE allows cells to grow to a higher OD_{600} over time. This could be due to the accumulated impact of slow proton leak through the functional transporter *or* it could be due to transport of some other metabolite or compound in the media that is a currently unidentified substrate of EmrE and whose transport has a small detrimental impact on *E. coli* growth. (c) Anti-His detection of leaky expression of WT- and E14Q-EmrE demonstrates that protein levels are equal for both constructs. Ponceau S stain of the western blot acts as a loading control. Similar results were obtained in three independent blotting experiments.

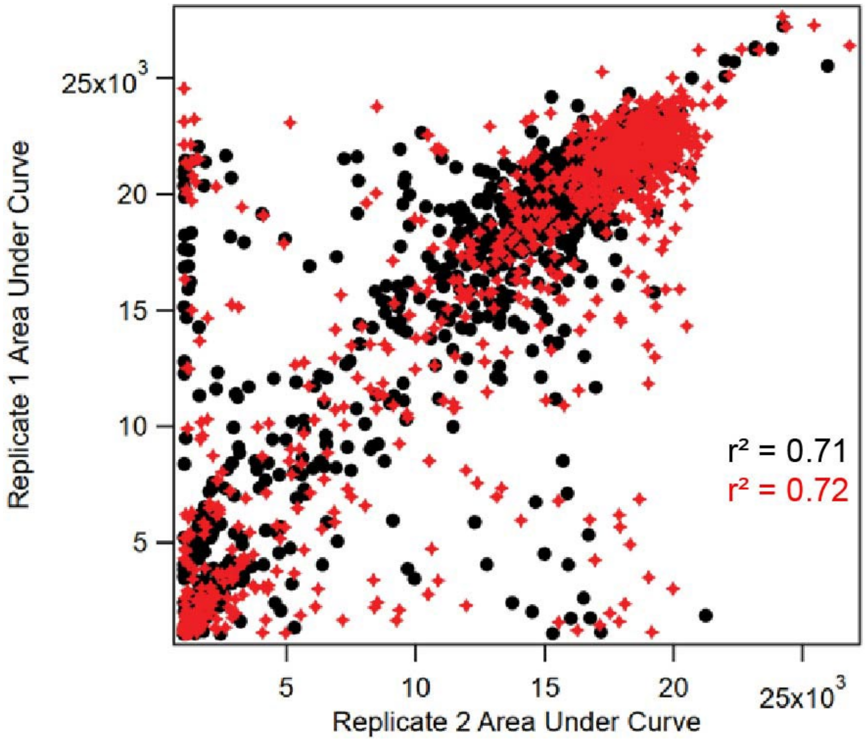

Supplementary Figure 2: Two independent Biolog replicates are strongly correlated. To quantify the correlation between replicates of the Biolog phenotype microarray, the area under the curve for all data in both biological replicates of the phenotype microarray for both WT-EmrE (black circles) and E14Q-EmrE (red diamonds) were plotted. The correlation constant for these data is 0.71 for WT (black) and 0.72 for E14Q (red). These data were the basis for the hit threshold determination for results from EmrE data. Divergence in the data may stem from plate-to-plate differences in initial OD of cells, final compound concentration, and volume of sample.

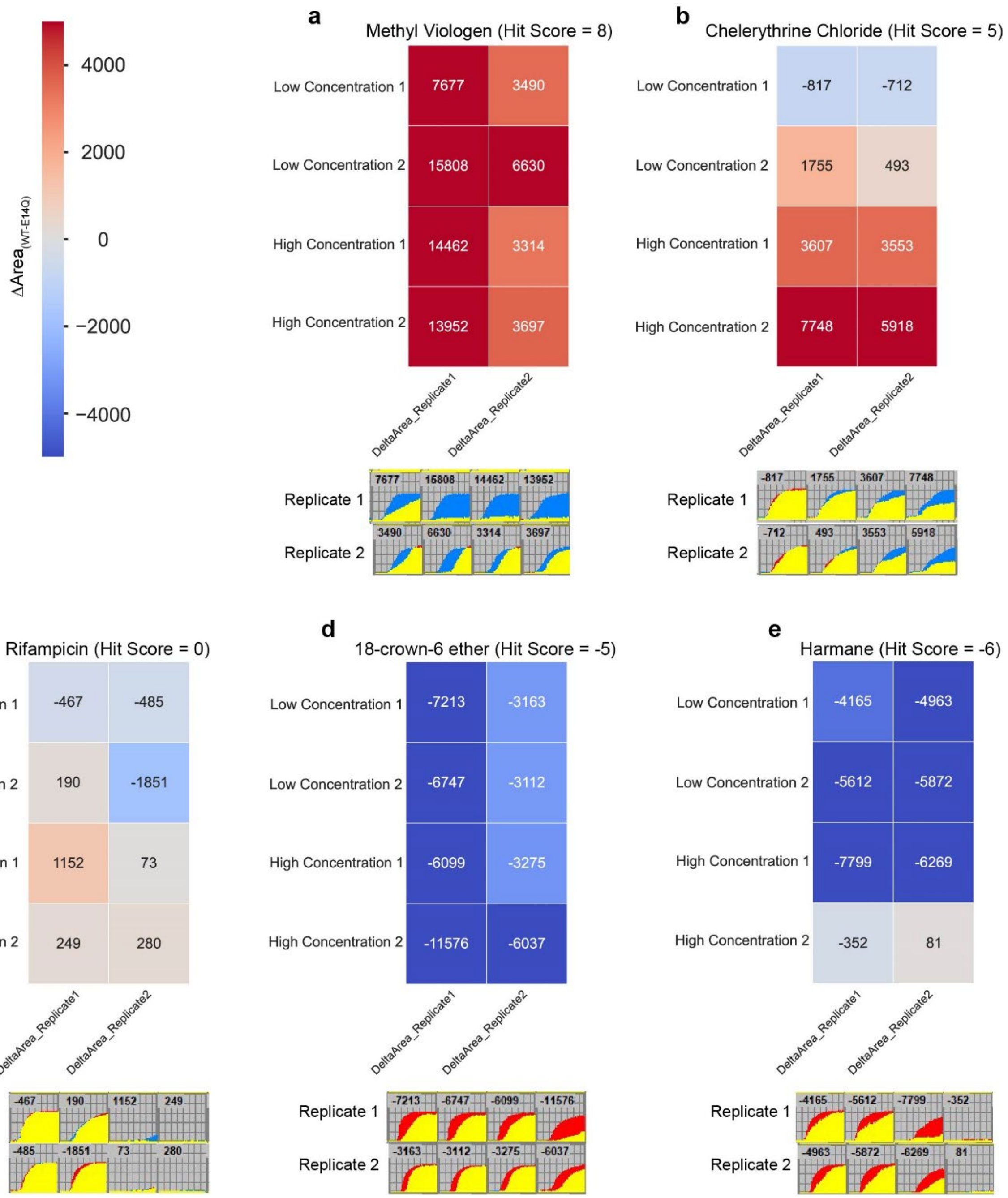

Supplementary Figure 3: Biolog Hit Scoring. Raw traces from the Biolog microarray (bottom panels labeled replicate 1 and 2) and calculated delta area under the curve (upper panels labeled high or low concentration 1 and 2) by well and by replicate for methyl viologen (a), chelerythrine chloride (b), rifampicin (c), 18-crown-6 ether (d), and harmane (e). Rifampicin is shown as an example of what the data look like for a non-hit in the context of this analysis. The color bar in the upper left shows the color coding for delta area spans the range used for hit selection following the criteria detailed in the Materials and Methods. For the raw microarray data, blue indicates WT-EmrE NADH production curves, red indicates E14Q-EmrE NADH production curves, and yellow shows overlap between WT- and E14Q-EmrE in this assay. Numerical ΔArea values are in the top right of each raw curve well, providing easy correspondence between the raw data and hit-score portion of the figures.

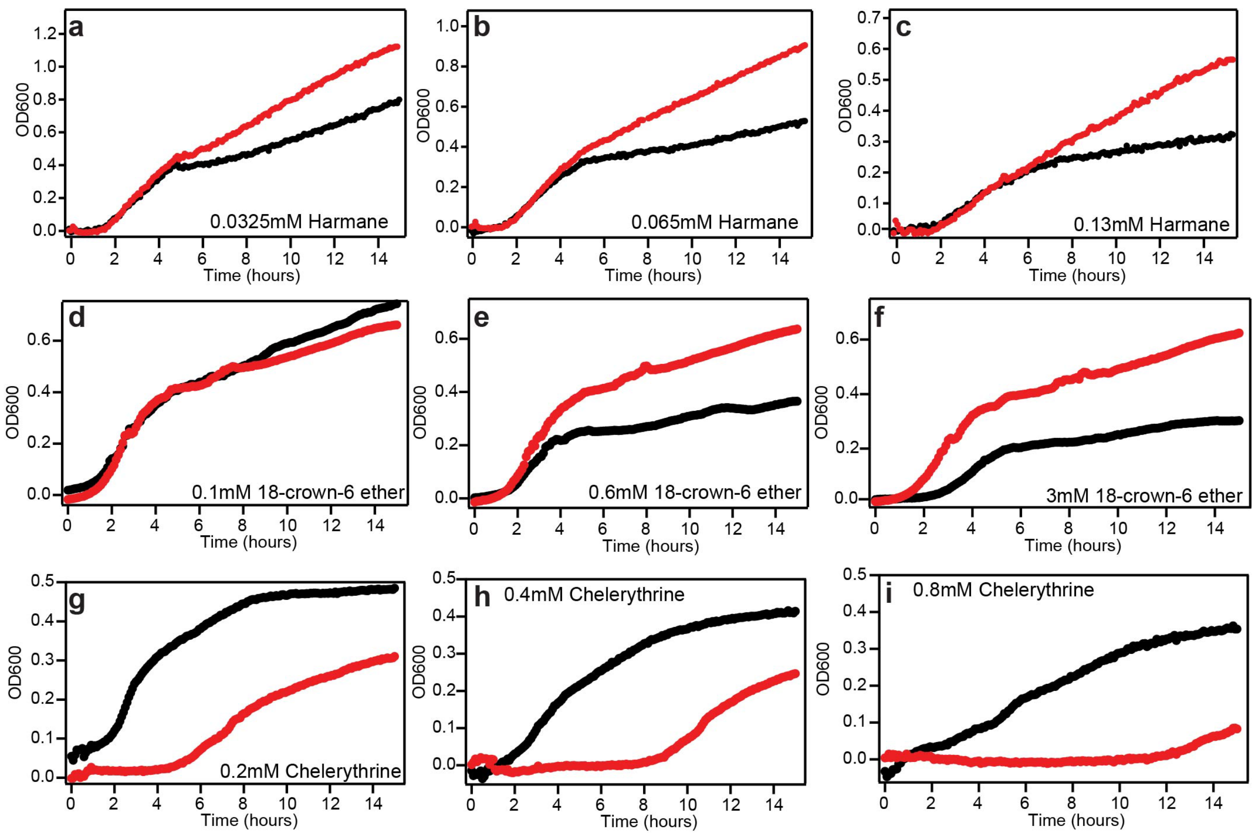

Supplementary Figure 4: Growth curve phenotypes are dose-dependent. To further validate the results of the Biolog phenotype microarray, growth curves at varying concentrations of harmane (A, 0.0325 mM; B, 0.065 mM; and C, 0.13 mM), 18-crown-6-ether (D, 0.1 mM; E, 0.6 mM; and F, 3 mM), and chelerythrine chloride (G, 0.2 mM; H, 0.4 mM; and I, 0.8 mM) were performed on MG1655 Δ*emrE* cells expressing WT-EmrE (black) or E14Q-EmrE (red). In all cases, the expected phenotypes (susceptibility for harmane and 18-crown-6-ether, and resistance for chelerythrine chloride) were observed with increasing compound concentration. Concentrations of compounds were selected based on the concentrations used in the Biolog phenotypic microarrays. The curves shown are an average of four biological replicates.

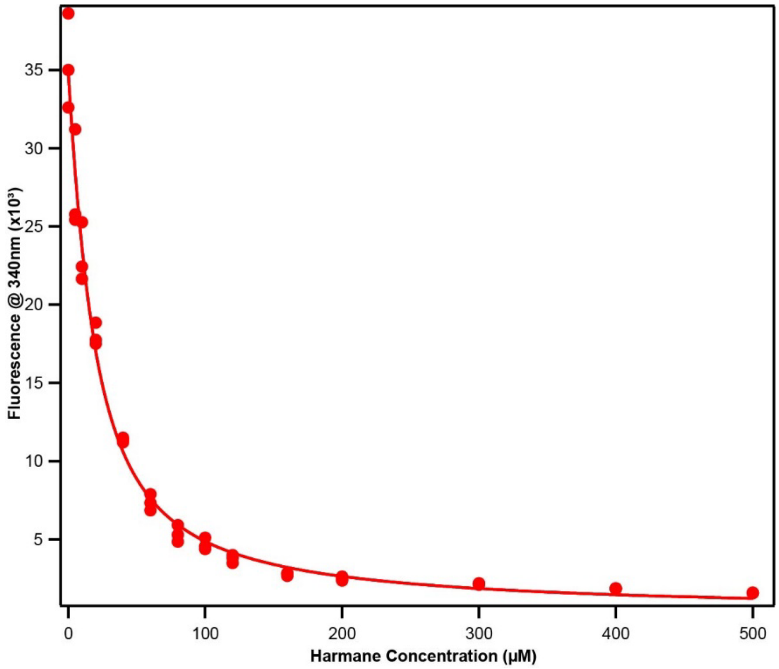

Supplementary Figure 5: Direct binding of harmane to S64V-EmrE. Harmane quenches intrinsic tryptophan fluorescence of S64V-EmrE in a dose-dependent manner, with an apparent K_d value of 14 ± 1μM.

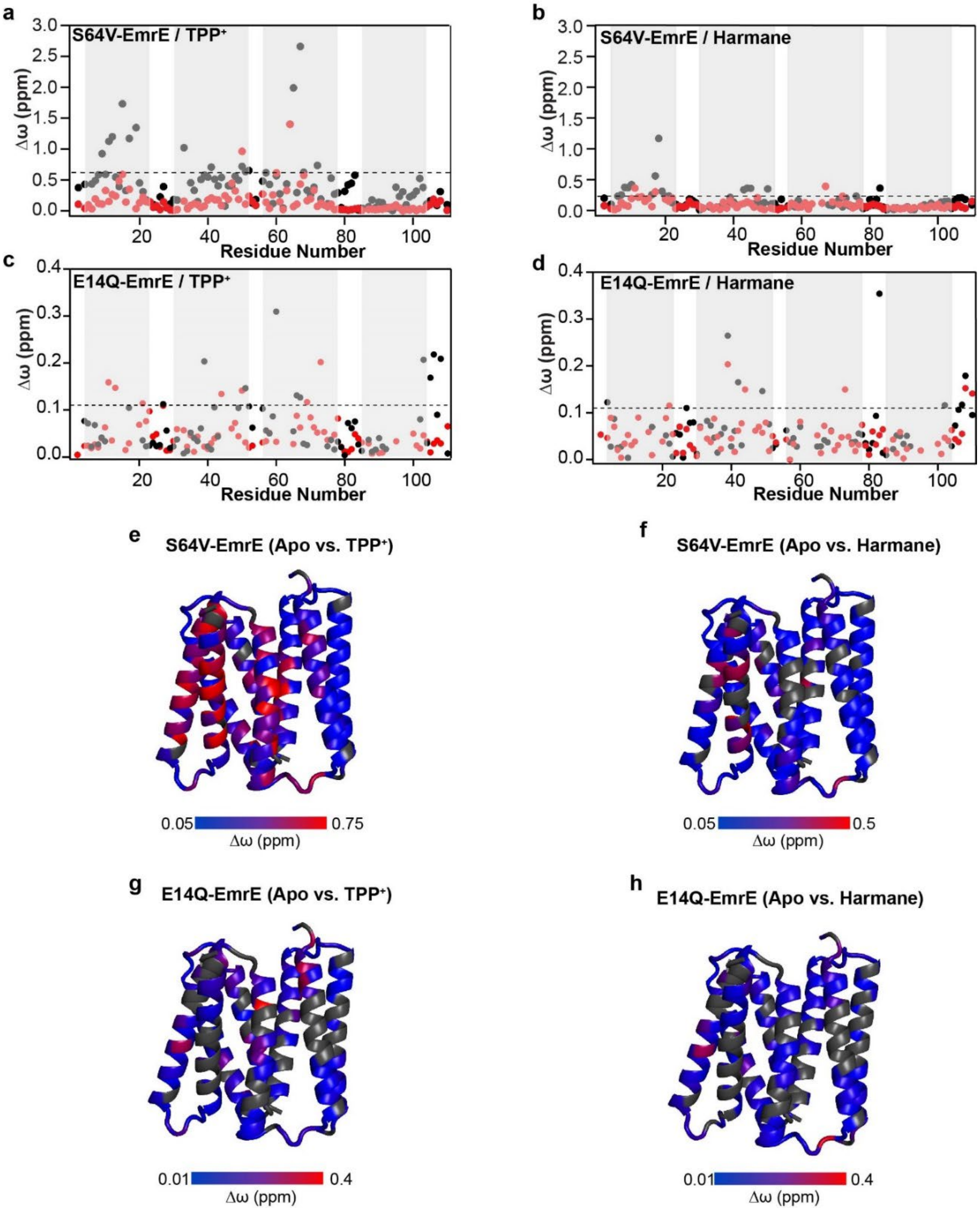

Supplementary Figure 6: Chemical Shift Perturbations upon harmane binding. NMR amide chemical shift perturbations (CSPs) upon TPP^+ and harmane binding to S64V-EmrE (a, b, respectively) and E14Q-EmrE (c, d, respectively) demonstrate direct binding of these compounds to EmrE. The data is also plotted on the structure for TPP^+ and harmane binding to S64V-EmrE (e, f, respectively) and E14Q-EmrE (g, h, respectively), Removal of the primary binding site in the E14Q-EmrE mutant diminishes the large-scale conformational change induced upon TPP^+ binding leading to smaller magnitude and less widespread CSP upon TPP+ binding to E14Q-EmrE compared to S64V-EmrE. Harmane appears to bind to the loop and tail regions in both S64V-EmrE and E14Q-EmrE.

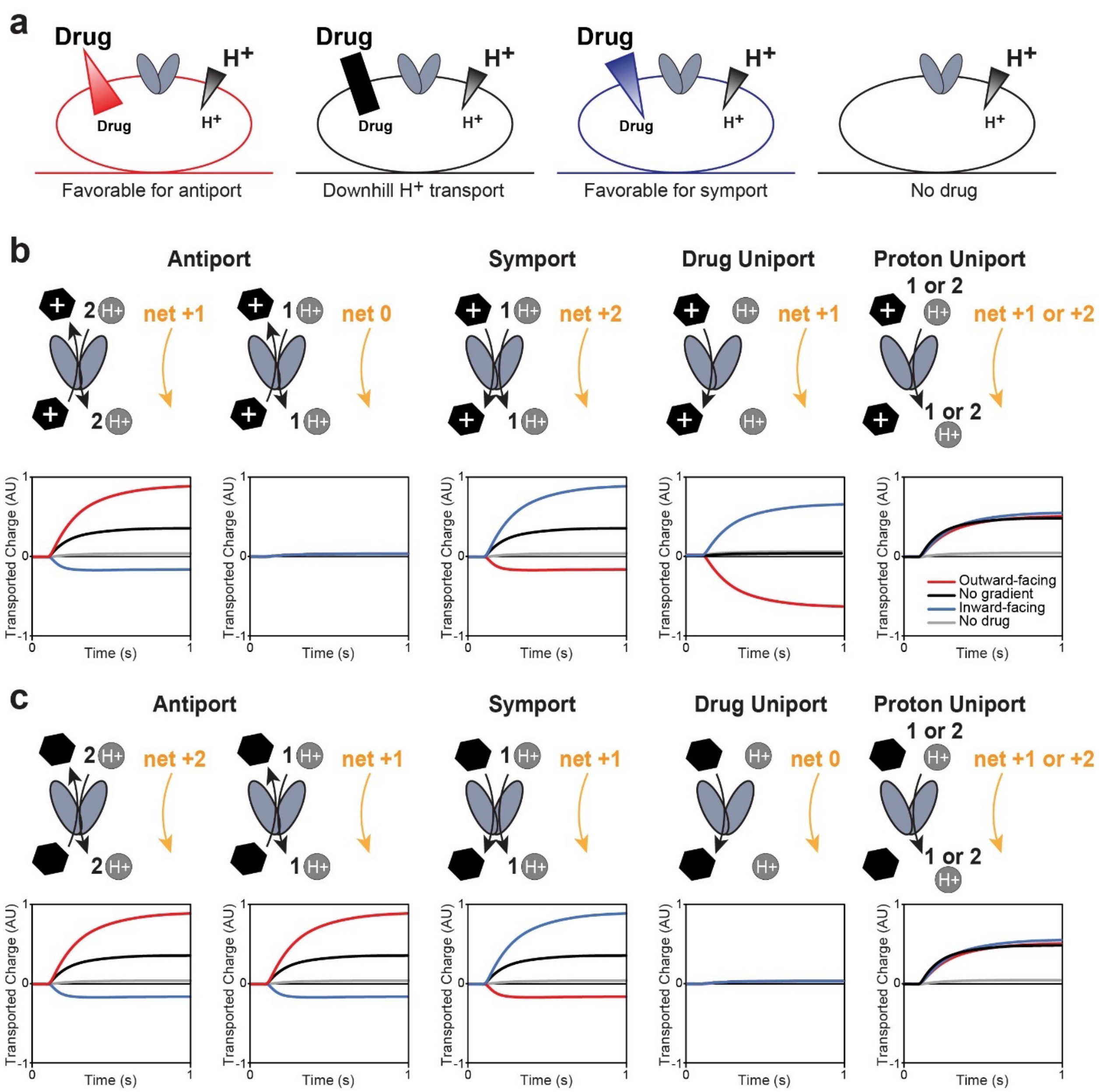

Supplementary Figure 7: Expanded SSME Expected Outcomes. This figure provides a more complete set of expected results for the SSME assay under the different assay conditions shown in (A). Predicted results for each possible coupled or uncoupled transport mode is shown for (B) +1 charged substrates and (C) neutral substrates, since these substrate species are explicitly studied in this manuscript. The expected outcomes for coupled 2 H^+ : 1 drug antiport, coupled 1 H^+ : 1 drug antiport, coupled 1:1 symport (note that there is no evidence of two protons binding simultaneously with a drug substrate as would be required for 2:1 symport), uncoupled drug uniport, and uncoupled proton uniport are shown for each condition. In the case of proton uniport, the predicted outcome is for substrate-gated proton uniport. The yellow arrow denotes the net charge moved per transport cycle in the given transport condition.

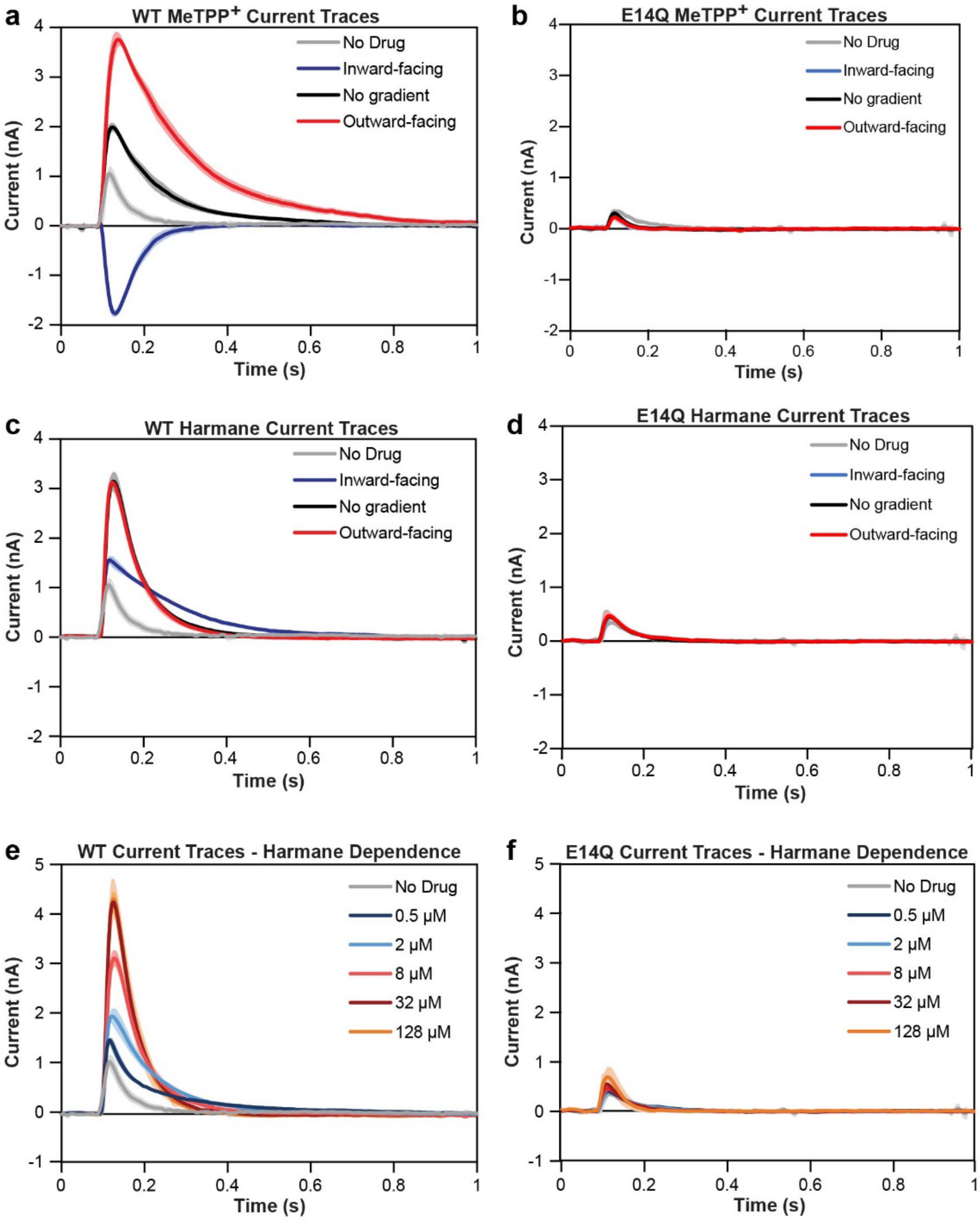

Supplementary Figure 8: Raw current traces for SSME data. Data shown is an average of three replicates, with standard deviation indicated by the shaded region. With both substrates, the minimal current is observed for sensors prepared using the non-functional mutant E14Q-EmrE (b, d, and f). $MeTPP^+$ (a and b) behaves as expected for an antiported substrate, with an increased signal when the drug and proton gradients are oriented in opposite directions and a reversal of transport direction when the large $MeTPP^+$ gradient is oriented in the same direction as the smaller proton gradient. Harmane (c and d) increases the transport signal compared to a background without drug. The signal is in the direction of downhill proton transport, regardless of the direction of the harmane gradient. Peak current of downhill proton transport increases in WT-EmrE proteoliposomes (e) with increased harmane concentration. E14Q-EmrE proteoliposomes do not show the same dependence on harmane concentration (f).

It is important to note that this assay also exclude dissipation of ΔpH by harmane diffusion through the membrane in an EmrE-independent manner. Harmane is hydrophobic and we must exclude the possibility that harmane can diffuse across the membrane in the neutral form and then re-equilibrate between protonation states, effectively acting to dissipate the pH gradient in the same manner that small

molecules like bicarbonate can dissipate the pH gradient. There is no detectable signal in the SSME assays using E14Q-liposomes (d, f), so if harmane can dissipate ∆pH in by diffusing through the membrane independent of EmrE it is not detectable by SSME. Furthermore, if harmane could non-specifically dissipate ∆pH in this manner, then in Supplementary Fig. 7a the condition with *only* a pH-gradient and equal drug concentration on both sides there would be no gradient if ∆pH is dissipated. This would result in no net driving force for transport, and no signal should be observed in Fig. 5c, d (main text), and Supplementary Fig. 7c for the black traces. This is not the case - transport still occurs in the same direction and to the same extent when driven solely by ∆pH indicating that the pH gradient remains intact. These *in vitro* results cleanly establish that harmane triggers proton leak through EmrE and not through a non-specific process through the membrane.

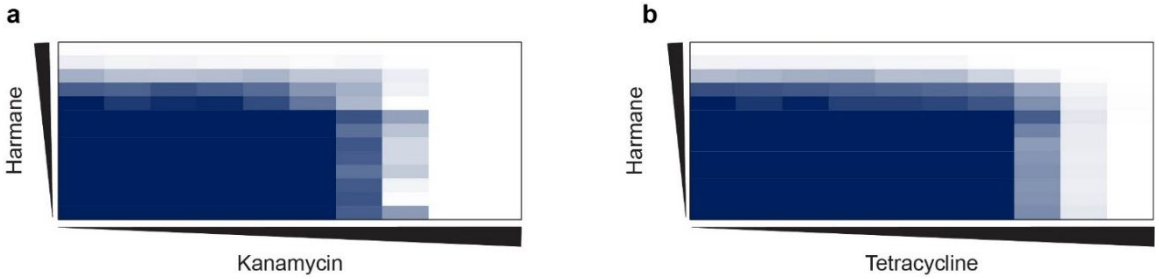

Supplementary Figure 9: E14Q-EmrE checkerboard assays. a) Checkerboard map of E14Q-EmrE expressing cells co-treated with harmane and kanamycin. b) Checkerboard map of E14Q-EmrE expressing cells co-treated with harmane and tetracycline. Concentration gradients are denoted by the black triangles. Growth is shown as a gradient of highest OD_{600} (navy) to no growth (white).

Supplementary Table 1: List of Biolog hits displaying resistance or susceptibility phenotypes.

Compound	Hit Score	Formal Charge	Notes
Harmane	-6	0	Specific reversible inhibitor of monoamine oxidase A
Hexachlorophene	-6	0	Antifungal, antiseptic
Menadione	-5	0	Vitamin K
18-crown-6-ether	-5	0	Metal-binding
Cefoperazone	-4	0	Cephalosporin antibiotic
Nitrofurazone	-4	+1	Antibiotic, interference of DNA synthesis
Oxytetracycline	-4	0	Antibiotic
Cobalt (II) chloride	-4	0	metal
Spectinomycin	-4	0	Antibiotic
Ethionamide	-4	0	Prodrug antibiotic,
Rolitetracycline	-3	0	Antibiotic, protein synthesis inhibition
Geneticin disulfate	-3	0	Antibiotic, protein synthesis inhibition
Ruthenium red	-3	0	Inorganic dye
Antimony (III) chloride	-3	0	Metal for vitamin A detection
Troleandomycin	-3	0	Macrolide antibiotic
Cefoxitin	-3	0	Cefalosporin antibiotic
Coumarin	-3	0	Metabolite
Nickel chloride	-3	0	Metal
Oleandomycin	-3	0	Macrolide antibiotic
Erythromycin	-3	0	Macrolide antibiotic
Dodine	-3	0	Fungicide
Glycine HCl	-3	0	Non-essential amino acid
Spiramycin	-3	0	Macrolide antibiotic
Manganese (II) chloride	3	0	Metal
Methyltrioctylammonium chloride	3	+1	Phase transfer catalyst
FCCP	3	0	Proton ionophore
Tetrazolium violet	3	+1	Dye, apoptosis inducer, antineoplastic agent
Cetylpyridinium chloride	4	+1	Antiseptic, destabilizes cell membranes
Acriflavine	4	+1	Local antiseptic, biological stain
Sanguinarine chloride	4	+1	Toxic polycyclic ammonium ion
Proflavine	4	0	Bacteriostat
Chelerythrine chloride	5	+1	Antibiotic, apoptosis induction
Crystal violet	6	+1	Topical antibiotic
Methyl viologen	8	+2	Herbicide, desiccant, photosystem-I inhibitor

Supplementary Table 2: Buffer Conditions for SSME experiments.

**Drug Gradient**	**Internal Buffer**	**External Buffer**
Inward-facing	0.5 μM drug<br>50 nM H^+ (pH 7.3)	8 μM drug<br>100 nM H^+ (pH 7.0)
Outward-facing	8 μM drug<br>50 nM H^+ (pH 7.3)	0.5 μM drug<br>100 nM H^+ (pH 7.0)
No gradient	8 μM drug<br>50 nM H^+ (pH 7.3)	8 μM drug<br>100 nM H^+ (pH 7.0)
No drug	50 nM H^+ (pH 7.3)	100 nM H^+ (pH 7.0)

Uncropped blots from Supplementary Fig. 1:
Anti-His Western Blot:

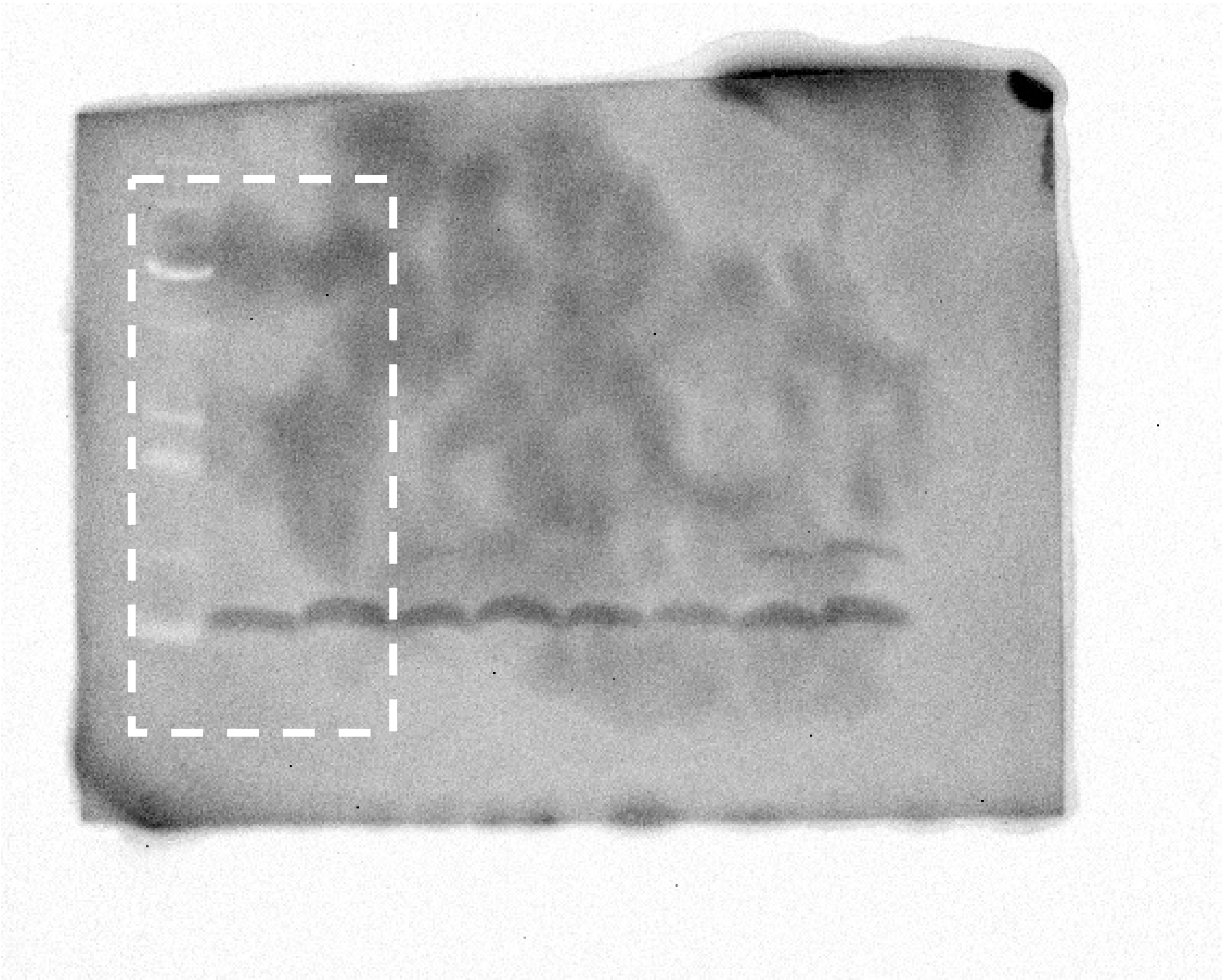

Ponceau S Loading Control:

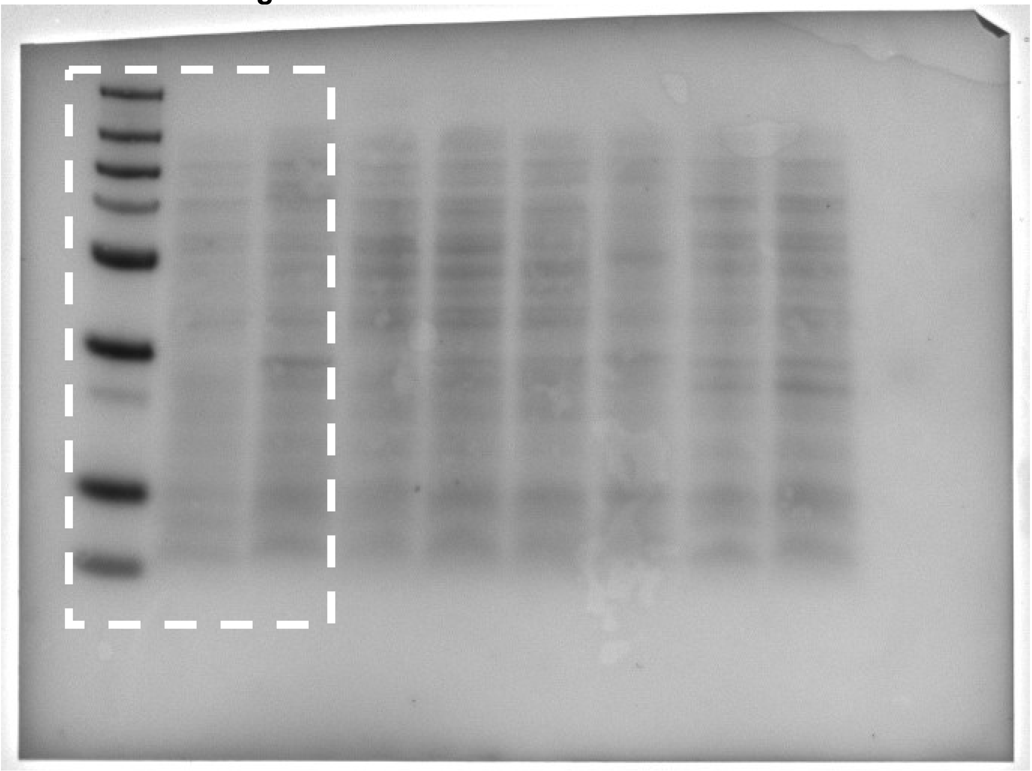

Supplement: Supplementary file 1 — Supplementary Information [file 41467_2022_35410_MOESM1_ESM.pdf]
