# Supplementary material for: Activating alternative transport modes in a multidrug resistance efflux pump to confer chemical susceptibility
Source: Nat Commun. 2022 Dec 10;13:7655. doi: 10.1038/s41467-022-35410-2 (PMC9741644; doi:10.1038/s41467-022-35410-2)
Supplement: Supplementary file 2 — Reporting Summary [file 41467_2022_35410_MOESM2_ESM.pdf]

nature portfolio

Corresponding author(s): Katherine Henzler-Wildman

Last updated by author(s): Nov 28, 2022

Reporting Summary

Nature Portfolio wishes to improve the reproducibility of the work that we publish. This form provides structure for consistency and transparency in reporting. For further information on Nature Portfolio policies, see our Editorial Policies and the Editorial Policy Checklist.

Statistics

For all statistical analyses, confirm that the following items are present in the figure legend, table legend, main text, or Methods section.

n/a	Confirmed	
☐	☒	The exact sample size (*n*) for each experimental group/condition, given as a discrete number and unit of measurement
☐	☒	A statement on whether measurements were taken from distinct samples or whether the same sample was measured repeatedly
☒	☐	The statistical test(s) used AND whether they are one- or two-sided<br>*Only common tests should be described solely by name; describe more complex techniques in the Methods section.*
☒	☐	A description of all covariates tested
☒	☐	A description of any assumptions or corrections, such as tests of normality and adjustment for multiple comparisons
☐	☒	A full description of the statistical parameters including central tendency (e.g. means) or other basic estimates (e.g. regression coefficient) AND variation (e.g. standard deviation) or associated estimates of uncertainty (e.g. confidence intervals)
☒	☐	For null hypothesis testing, the test statistic (e.g. *F*, *t*, *r*) with confidence intervals, effect sizes, degrees of freedom and *P* value noted<br>*Give P values as exact values whenever suitable.*
☒	☐	For Bayesian analysis, information on the choice of priors and Markov chain Monte Carlo settings
☒	☐	For hierarchical and complex designs, identification of the appropriate level for tests and full reporting of outcomes
☒	☐	Estimates of effect sizes (e.g. Cohen's *d*, Pearson's *r*), indicating how they were calculated

Our web collection on statistics for biologists contains articles on many of the points above.

Software and code

Policy information about availability of computer code

Data collection	NMR data collected on Bruker spectrometers was collected using Topspin (V.3.5pl7).
Data analysis	NMR data was processed using nmrPipe (V 11.1) and and spectra were visualized using ccpNmr analysis(V 3.0). Reconstruction of non-uniformly sampled NMR data was performed using the SMILE algorithm implemented in nmrPipe (V 11.1). Other data were analyzed using Excel (2019) and IGOR Pro (V 7.0.8.1).

For manuscripts utilizing custom algorithms or software that are central to the research but not yet described in published literature, software must be made available to editors and reviewers. We strongly encourage code deposition in a community repository (e.g. GitHub). See the Nature Portfolio guidelines for submitting code & software for further information.

Data

Policy information about availability of data

All manuscripts must include a data availability statement. This statement should provide the following information, where applicable:

- Accession codes, unique identifiers, or web links for publicly available datasets
- A description of any restrictions on data availability
- For clinical datasets or third party data, please ensure that the statement adheres to our policy

The raw and analyzed datasets generated in this study are available in the MendeleyData repository, https://doi.org/10.17632/tpcdgw7h6m.1. NMR chemical shift data can be found on the Biological Magnetic Resonance Data Bank (BMRB) under the following accession numbers: 51024, 51025, 51026, 51027, 51030, 51031 [https://bmrb.io/]. Raw NMR data was deposited on the BMRbig database under the following accession numbers: BMRbig18, BMRbig19, BMRbig20, BMRbig21,

BMRbig22, and BMRbig23 [https://bmrbig.bmrb.io/]. Full gel images of western blots are supplied in the Supplementary Data/Source Data file.

Human research participants

Policy information about studies involving human research participants and Sex and Gender in Research.

Reporting on sex and gender	not applicable
Population characteristics	not applicable
Recruitment	not applicable
Ethics oversight	not applicable

Note that full information on the approval of the study protocol must also be provided in the manuscript.

Field-specific reporting

Please select the one below that is the best fit for your research. If you are not sure, read the appropriate sections before making your selection.

[x] Life sciences [ ] Behavioural & social sciences [ ] Ecological, evolutionary & environmental sciences

For a reference copy of the document with all sections, see nature.com/documents/nr-reporting-summary-flat.pdf

Life sciences study design

All studies must disclose on these points even when the disclosure is negative.

Sample size	The number of replicated data points for all relevant experiments were determined based on the field standards for the given experiment. A number of papers using similar techniques were surveyed and the average sample sizes were taken into account to determine our sample sized.
Data exclusions	No data was excluded from this work.
Replication	All experiments were replicated successfully three times.
Randomization	This study does not require randomization because we are using internal controls to a non-functional mutant of our transporter for the cellular assays.
Blinding	Blinding is not required for this study because the researchers must know the details of each sample in order to interpret the data and we use a comparison of functional and non-functional transporter in both in vivo and in vitro experiments.

Reporting for specific materials, systems and methods

We require information from authors about some types of materials, experimental systems and methods used in many studies. Here, indicate whether each material, system or method listed is relevant to your study. If you are not sure if a list item applies to your research, read the appropriate section before selecting a response.

Materials & experimental systems

n/a	Involved in the study	
[x]	[ ]	Antibodies
[x]	[ ]	Eukaryotic cell lines
[x]	[ ]	Palaeontology and archaeology
[x]	[ ]	Animals and other organisms
[x]	[ ]	Clinical data
[x]	[ ]	Dual use research of concern

Methods

n/a	Involved in the study	
[x]	[ ]	ChIP-seq
[x]	[ ]	Flow cytometry
[x]	[ ]	MRI-based neuroimaging
